# Expression signatures with specificity for type I and II IFN response and relevance for autoimmune diseases and cancer

**DOI:** 10.1186/s12967-025-06628-7

**Published:** 2025-07-03

**Authors:** Bogac Aybey, Benedikt Brors, Eike Staub

**Affiliations:** 1 Clinical Measurement Sciences, Merck Healthcare KGaA, Oncology Data Science, Darmstadt, Germany; 2 Faculty of Biosciences, Heidelberg University, Heidelberg, Germany; 3 Division of Applied Bioinformatics, German Cancer Research Center (DKFZ), Heidelberg, Germany; 4 German Cancer Consortium (DKTK), German Cancer Research Center (DKFZ), Heidelberg, Germany; 5 Medical Faculty Heidelberg, Faculty of Biosciences, Heidelberg University, National Center for Tumor Diseases (NCT), Heidelberg, Germany

**Keywords:** Immunology, Interferon, Gene expression signature discovery, Transcriptomics, SLE-systemic lupus nephritis, Oncology

## Abstract

**Background:**

Aberrant interferon signaling is a key element of various diseases, but resolving gene expression signatures that stem from different types of IFNs in tissue samples is still a challenge. Most published IFN signatures comprise genes that are activated by different IFNs: they cannot discriminate type-I (IFN-I) and type-II (IFN-II) IFN stimulation. Most often such signatures were obtained from a single expression dataset that had been obtained in a specific cellular context, and their translatability to other experimental contexts has not been demonstrated.

**Results:**

We leveraged multiple RNA-seq datasets of IFN stimulation in a network meta-analysis workflow to obtain IFN gene signatures separating IFN-I and IFN-II. We validated our signatures in bulk and single cell RNA-seq datasets of various cellular contexts demonstrating similar or higher coherence than previously published signatures. Our IFN-II signature is broader applicable than other published signatures as it demonstrates strong performance in detecting IFN-II response in more cell types. In three SLE microarray datasets our IFN-I signature was highly coherent and correlated with disease severity better than most published signatures. In TCGA, our IFN-II signature produced distinct profiles compared to published IFN-I signatures and correlated strongly with published CD8^+^ T cell signatures. In cohorts of three different cancer types, we observed higher signature scores of our IFN-II signature in responders than in non-responders to immune checkpoint inhibitor (ICI) therapy.

**Conclusions:**

Our IFN-I and IFN-II response-specific gene expression signatures can inform on complex IFN responses in a more fine-grained way than previously possible. They can be used to assess type I versus II IFN response in gene expression data produced by different technologies, for different diseases and even different cell types in single cell studies. The association of high scores of our IFN-II signature with anti-tumor response to ICIs suggests a role as a biomarker to predict ICI response.

**Supplementary Information:**

The online version contains supplementary material available at 10.1186/s12967-025-06628-7.

## Introduction

Interferons (IFNs) are a group of cytokines that elicit different transcriptional responses, which vary depending on the type of IFN. Type-I IFN (IFN-I) and type-III IFN (IFN-III) share the same downstream signaling pathway whereas type-II IFN (IFN-II) uses different downstream signaling elements. IFN-I and IFN-III utilize the JAK-STAT signaling pathway involving JAK1, STAT1 and STAT2 that is ultimately leading to formation of STAT1-STAT2-IRF9 complexes [1]. These activate interferon stimulated response elements on genomic DNA which leads to induction of many interferon-stimulated genes (ISGs). IFN-II signaling induces ISGs through a distinct pathway involving JAK1, JAK2 and STAT1 which leads to the formation of STAT1-STAT1 homodimers which activate gamma-activated sequences [2]. For the various IFN-I ligands (α, β, ε, ω and κ) many reports have proposed differing downstream transcriptomic effects [1, 3–5].

A multitude of IFN gene expression signatures, i.e. sets of IFN-regulated genes that yield an IFN signature score per sample, have been published: some with claims to be specific for IFN-I vs. IFN-II, many of them being disease-, treatment-, cell type- or dataset-specific [6–10]. Especially in the clinical setting, the status of IFN signaling is usually determined indirectly by the expression of IFN signatures consisting of multiple ISGs [11]. The published signatures are most often formed by ISGs of both, IFN-I, and IFN-II. Most signatures usually do not differentiate between different IFN types [12] and are dominated by IFN-I ISGs. They are often not widely tested in original studies, and hence often not applicable across multiple cellular environments, disease contexts and studies. These drawbacks limit the interpretation of the specific transcriptional effects of IFN types in heterogeneous cell populations of complex tissues.

Distinct signaling by different types of IFNs plays a crucial role in various diseases and their therapies, such as in cancer [13] and in auto-immune diseases like systemic lupus erythematosus (SLE) [14]. In SLE, early gene expression studies found that elevated IFN-I signaling is associated with the SLE disease activity index (SLEDAI) which reflects disease severity [15]. Since then, several IFN signatures have been discovered leveraging SLE datasets to examine ISGs related to different parameters of the disease [7, 8]. In many gene expression signature analyses of clinical cancer samples, various types of IFN signatures represent a group of signatures that usually cluster well together to the exclusion of other signatures and therefore represent robust transcriptomic signals in cancer [16]. Recently, a six-gene IFN-II based gene expression signature derived from multiple cancer anti-PD-L1 trial datasets showed that responders had higher IFN-II signature scores at therapy baseline [6]. Similarly, elevated IFN-II gene expression scores or expression of IFN-II response genes at baseline as an ICI response marker have been also reported by others [17–19]. These examples highlight the importance of understanding the role of responses to different IFNs in disease pathogenesis and their utility for the prediction of treatment outcomes.

Despite these advances in understanding of differential gene regulation by IFN-I and IFN-II, the detection of these differences in complex tissue samples in a clinical context is often not possible. A comprehensive analysis contrasting multiple published datasets is lacking to better understand which differences in transcriptional programs are elicited by different IFNs and whether they can be reliably detected in different clinical contexts.

Therefore, in this study our aim was to determine if it is possible to differentiate the signals of the commonly used IFN-I ligands, IFN-α (IFN-a) and IFN-β (IFN-b), and if we could identify gene modules to better distinguish signals from IFN-I and IFN-II. To this end, we conducted a meta-analysis of five IFN stimulation bulk tissue RNA-seq datasets of various healthy cellular environments to obtain distinctive IFN-I and IFN-II gene expression signatures. We evaluated our signatures in comparison to known IFN signatures. We further investigated the relevance of our signatures in diseases and therapies in three SLE gene expression datasets and in cancer gene expression datasets of TCGA cohorts and of ICI studies in three cancer types.

## Data and methods

### Datasets and processing

We downloaded raw expression matrices for bulk tissue IFN stimulation RNA-seq datasets from public repositories: 1st donor and BEAS-2B cell line from Ziegler dataset (via contact with the authors), Jankowski dataset (Gene Expression Omnibus (GEO) accession number GSE161916), Rai dataset (http://www.ilincs.org/apps/grein/?gse=GSE74863) and Lee dataset [20] (GEO accession number GSE161664) [21]. We normalized raw counts using *DESeq2* [22]. We obtained processed transcripts per kilobase million (TPM) matrices for Colli dataset (https://cmdga.org with GEO accession code GSE148058, GSE133218) and 2nd donor from Ziegler dataset (GEO accession number GSE148829). We obtained fragments per kilobase of transcript per million mapped reads (FPKM) matrix for Fujiwara dataset (GEO accession number GSE120844). We downloaded *DESeq2* normalized count matrix for Devlin dataset (GEO accession number GSE145647). We filtered out lowly expressed genes (genes with overall expression < 10 for raw counts and < 1 for normalized counts). We list the details of these datasets, separated by their use for signature discovery or validation, in Table 1.

**Table 1:** Datasets used in the discovery and validation steps

	Dataset	Cell source	Stimulation	Dose [ng/ml]	Time [h]
**Discovery**	[23] Donor 1	Lung-Basal cells	IFN-a, IFN-g	0.1, 0.5, 1, 2, 5, 10	12
	[24]	Kidney-HPPT cell line	IFN-a, IFN-b, IFN-g	10	12
	[25]	Skin-SK-MEL-624 cells	IFN-g	10	24
	[26]	Pancreas-EndoC-BH1 cell line	IFN-a	11 pg/ml	2, 8, 18
	[27]	Lung-IMR90 cell line	IFN-b	10	24
**Validation**	[23] Donor 2	Lung-Basal cells	IFN-g, IFN-a	0, 0.1, 0.5, 1, 2, 5, 10	12
	[23]	Lung-Bronchial cell line (BEAS-2B)	IFN-g, IFN-a	0, 0.1, 0.5, 1, 2, 5, 10	12
	[28]	Whole Blood	IFN-b, IFN-g, IFNλ	2 (IFN-a), 10 (IFN-g & IFN-λ)	4
	[20]	Lung-Small airway epithelial cells (SAEC)	IFN-a, IFN-b, IFN-g, IFNλ	10	12

Datasets are given along their experimental conditions

For SLE microarray datasets, we obtained RMA (GEO accession numbers GSE121239 and GSE121239) or batch normalized gene expression matrices (GEO accession number GSE65391) only from baseline samples. We included SLEDAI values from each publication for each patient in our analysis.

We downloaded TCGA bulk tissue RNA-seq expression data from the Xena database [29] and used TPM-normalized values. For immune checkpoint inhibitor trial bulk tissue RNA-seq datasets of gastric cancer-pembrolizumab [30] and melanoma-ipilimumab [31], we obtained FPKM processed datasets from Cui et al. [32]. For IMvigor210 bladder trial with atezolizumab treatment, we downloaded raw gene expression data from http://research-pub.gene.com/IMvigor210CoreBiologies [33] and transformed into TPM values. We also included response data from the given sources in the analysis.

We assessed a single cell RNA-seq dataset of PBMCs stimulated with IFN-beta (IFN-b), IFN-gamma (IFN-g), and TNF-alpha (TNF-a) in our study. We downloaded raw counts of scRNA-seq GSE181897 dataset [34] from GEO (GEO accession number GSE181897). We analyzed the data using *Seurat* [35]. We removed cells expressing less than 200 genes or more than 4,000 unique gene counts and genes expressed in less than three cells. We filtered out cells having mitochondrial gene portion more than 5%. For downstream analyses, we normalized the raw expression values of each single cell dataset using NormalizeData() from *Seurat*. Additionally, to confirm our observations about IFN-g stimulation in non-myeloid cells, we analyzed another independent published temporal (1 h and 6 h) IFN-g stimulation PBMC scRNA-seq dataset by Kartha et al. [36]. We performed preprocessing of this scRNA-seq dataset as described previously [37]. We classified immune cell types based on our published random forest-based classification utilizing our immune type gene signatures [37]. As our gene features, we utilized 167 genes from our signatures for eight different cell type populations (plasma, monocytes, DC, pDC, B, NK, CD4^+^ and CD8^+^ T cells). As a reference PBMC dataset for the cell type classification, we used fine grained cell type annotations from a PBMC reference dataset- the Hao dataset [38]. For further analyses, we filtered out cell types (plasma, ILCs and double-negative T cells) with less than ten cells in each condition.

### Network meta-analysis workflow

We analyzed individual discovery datasets to identify differentially expressed genes (DEGs) following different IFN treatments. Prior to statistical analyses for identification of DEGs, we selected the common top 5,000 highly variable genes across all discovery datasets by summing up their ranks based on variance and prioritizing those 5,000 genes with the highest additive rankings for further analysis. Then, we performed statistical analyses for differential gene expression (DGE) on these 5,000 highly variable genes for each discovery dataset, separately. We used log_2_-transformed normalized expression values as input to linear models using *limma* [39]. The models were fitted to the data using lmFit function with different dose or time points as co-variates and the analysis was performed using the eBayes function (setting trend = T). We obtained estimated regression coefficients (interpreted as log-fold change (logFC)) and its standard errors from each comparison for each gene and discovery dataset for the subsequent analysis of discovering IFN type specific signatures.

**Fig. 1:**
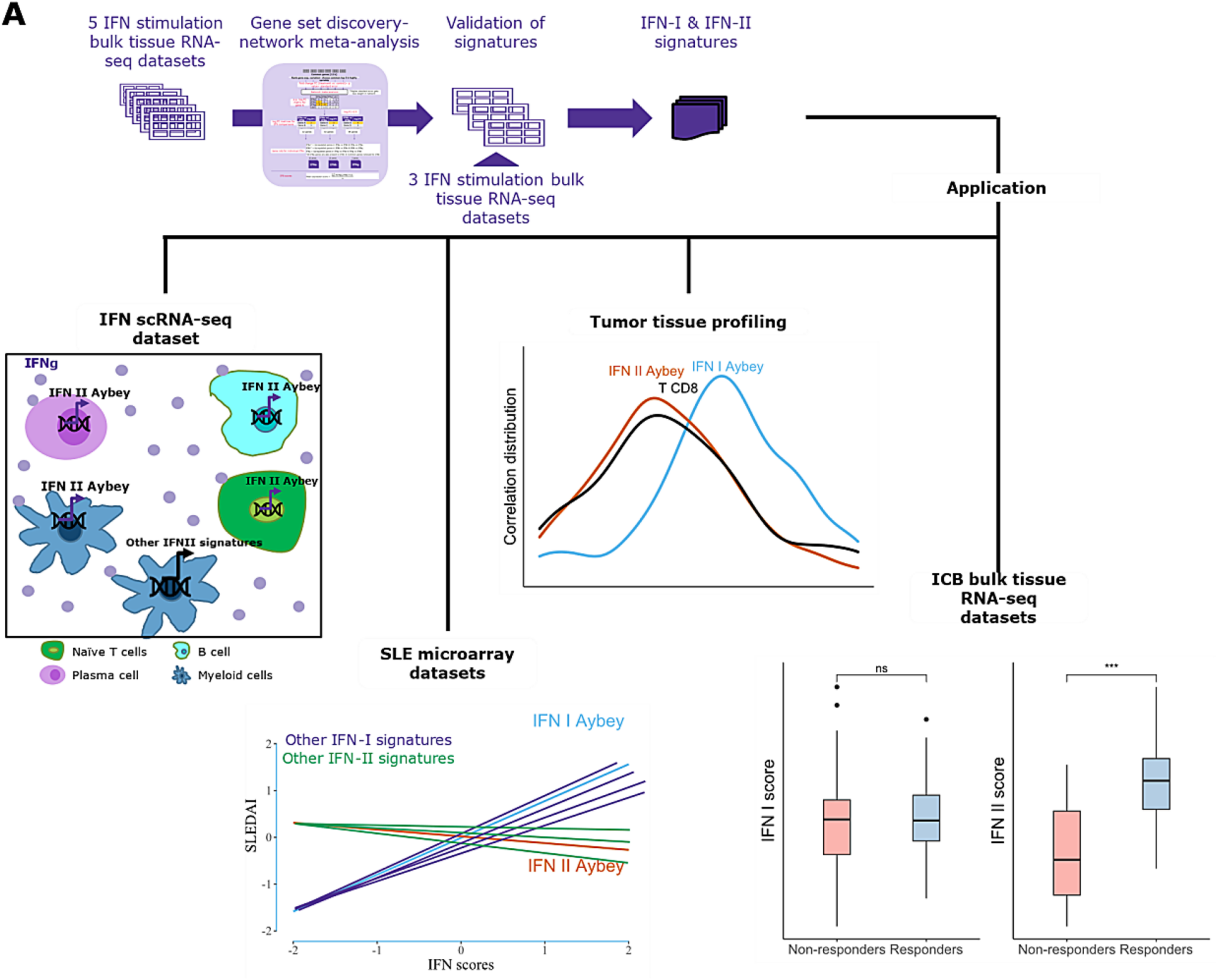

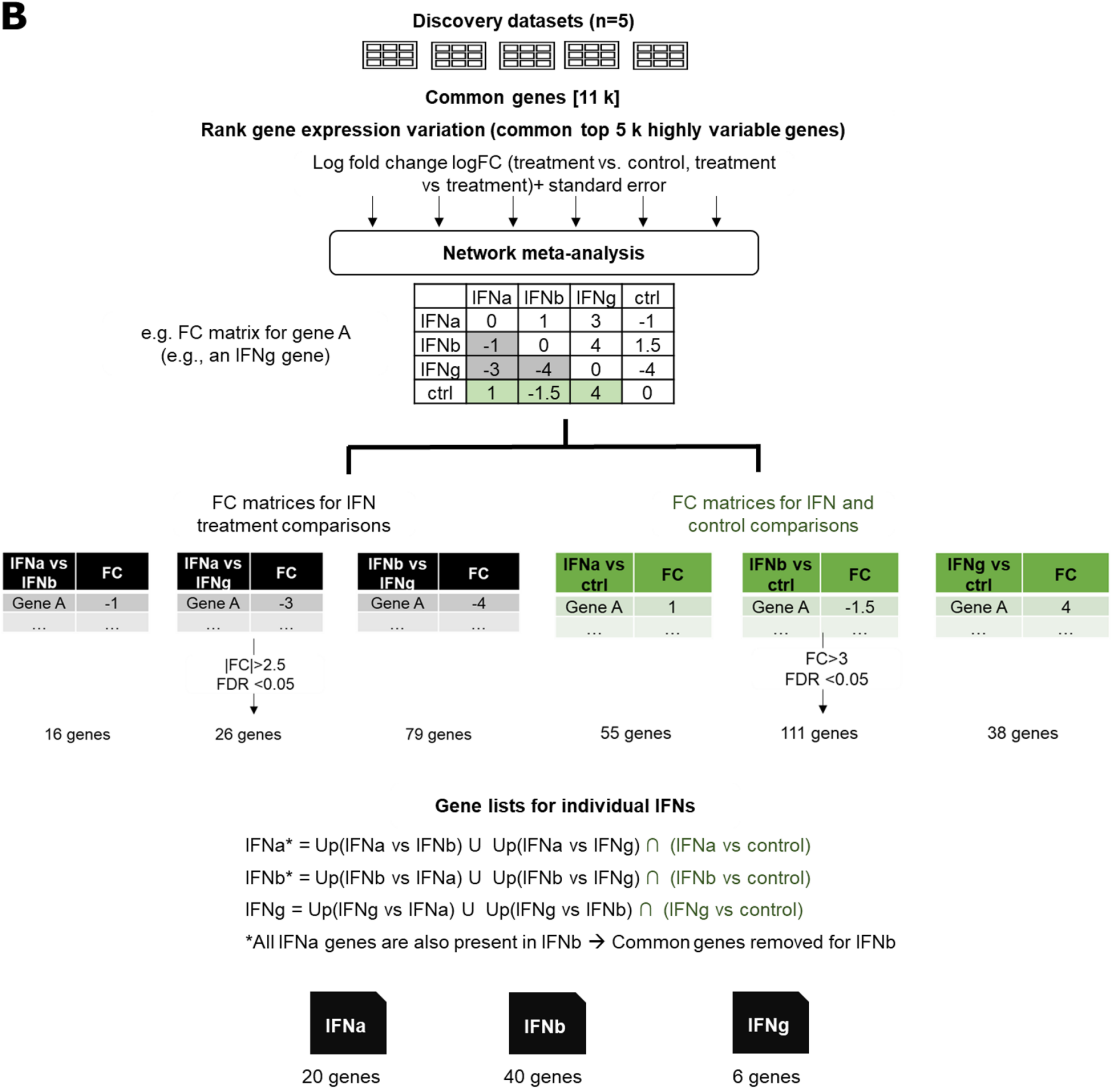
(**A**) Schema of our study. (**B**) Network meta-analysis workflow for obtaining IFN signatures. For the workflow, five bulk tissue RNA-seq IFN stimulation discovery datasets are used. ~11,000 common genes are ranked based on variance in each dataset. The common top 5,000 highly variable genes are selected for downstream analysis. Log fold changes (logFC) between stimulation and control, and between each available treatment are calculated for each dataset, along standard errors. Network meta-analysis is applied on these results to obtain FC values and FDR-adjusted p-values between each IFN treatment for each gene along with comparison between each IFN treatment and control (ctrl). Three lists of FC for IFN treatment comparisons are compiled and genes for |FC| less than 2.5 were filtered out. Additionally, three lists of FC for IFN treatment and control comparison are compiled and only genes with more than FC of 3 are taken into consideration. Finally, gene list for each individual type of IFN is constructed by taking only upregulated genes in each IFN type compared to other treatments or control

Utilizing network meta-analysis, we compared multiple treatments across various studies, extending traditional meta-analysis approaches as detailed in Rücker et al. (2012) [40] and Winter et al. (2019) [41]. Within this framework, treatments are represented as nodes within a network, and direct effects are computed akin to traditional meta-analysis methods using variance-weighted effect size averages. Given that not all treatments were uniformly represented across five datasets in our analysis, network meta-analysis allowed for the assessment of indirect comparisons that might not have been possible with traditional methods. By aggregating direct estimates along possible paths between treatments, indirect effect sizes complement direct estimates, increasing the reliability of comparisons across studies. For comparisons involving only one or two studies, effect estimates are supported by multiple other studies. Conversely, comparisons supported by multiple studies tend to yield effect estimates like those obtained through traditional meta-analysis techniques. Indirect and direct effect sizes are summed up for the calculation of overall effect estimates of individual comparisons and random effect model is applied to account for heterogeneity in different studies. For the implementation, we provided *limma* results as input to *netmeta* [42]. We used logFC between control and treatments along with standard errors as input. The output for each gene includes a 4 × 4 FC matrix along with p-values for each comparison. This matrix contains estimated FC values derived from comparisons among all conditions such as IFN-a vs. IFN-b, IFN-b vs. IFN-g, IFN-a vs. IFN-g, and the control against all three treatments (control vs. IFN-a, control vs. IFN-b and control vs. IFN-g) (Fig. 1B). The summary p-values accompanying the matrix address the null hypothesis that there is no significant difference in treatment effectiveness among the compared groups. To account for multiple correction errors and control the false discovery rate (FDR), we applied Benjamini-Hochberg correction to the raw p-values coming from each treatment comparison separately.

### Comparisons with published signatures: focus on type I/II IFN gene signatures

We compared our own type I/II IFN gene expression signatures to various published signatures. To this end, we obtained a curated list of published coherent high quality signatures for IFN signaling from our RosettaSX platform for signature evaluation [16]: IFNa-Hallmark [43], IFNg-Hallmark [43], IFN-Bilgic [44], IFN-Feng [45], IFN-MB-Staub [10], IFN-Rice [46], IFN-SLE-Bennett [7], IFN-Walsh [47], IFNg-Dummer [9], IFN-Chaussabel M1-2 [8], IFN-Chaussabel M3-4 [8] and IFN-Chaussabel M5-12 [8]. We also included further published IFN-II signatures in our comparative analysis: IFNg-Ayers [6], IFNg-Azizi-Platanias [48, 49], IFNg-Sharma [50] and IFNg-Waddell [51].

We calculated mean signature expression scores for each sample by averaging the z-scaled (across samples) log_2_-transformed normalized expression values e.g., log_2_(TPM + 1) of all genes for a given signature. To determine the relevance of the signatures in an expression dataset, we calculated Coherence Scores [10]. The Coherence Score is based on the mean of Pearson correlation of all gene pairs in the gene signature in a specific dataset. It varies between − 1 and + 1 defining weak and strong correlation between the genes in the signature, respectively. Coherence scores > 0.2 generally identify signatures that are coordinately regulated transcriptional modules in a dataset, and therefore point to good translatability into and relevance in a new dataset [16].

To examine the separability of our IFN type genes, we reduced the dimensionality of GSE181897 scRNA-seq dataset of control, IFN-b and IFN-g treatment samples using UMAP (2 principal components) based on either our or published IFN signature genes.

### Evaluation and comparison of our signatures and their relationship to disease parameters

For our study, we employed both Pearson and Spearman correlation methods to capture different types of relationships in our data.

We applied Pearson correlation in the analyses comparing signature scores among TCGA cohorts, since it is reasonable to assume that the signature scores are normally distributed and we are interested in their linear relationship. For each TCGA cohort, we calculated Pearson correlation between each published IFN-I or CD8^+^ T cell signatures (Charoentong [52], Angelova [53], Becht [54], Bindea [55], Nieto [56], Newman [57], and Aybey [37]) with each other separately or with our signatures. We transformed correlation coefficients into Z-scores using Fisher’s transformation, with the advantage that these are normally distributed and better suited for statistical testing.

We used Spearman correlation to examine the association between signature expression scores and SLEDAI values, as those datasets exhibited non-normal skewed distributions with potential outliers (see Supp. Figure 1). To examine the monotonic relationship of the gene signatures to the SLE disease parameters, we calculated Spearman correlation between mean signature expression scores and SLEDAI.

For hypothesis testing of the differences between distributions of Z-transformed correlation scores or differences between mean signature expression scores in responders and non-responders or of our IFN signatures we used two-sided Student’s t-test.

To evaluate the predictive performance of the IFN-II-Aybey signature compared to several published IFN signatures, we performed receiver operating characteristic (ROC) curve analyses on ICI therapy cohorts. For each patient sample, we calculated the mean signature scores for each IFN signature. We dichotomized patients into responders and non-responders based on the clinical outcome data available for each ICI cohort. Logistic regression models were subsequently fitted using the signature scores as predictors. The area under the ROC curve (AUC) was calculated for each signature using the predicted probabilities on *pROC* package in R. This approach provided a quantitative metric to directly compare the performance of IFN signatures in predicting ICI therapy response.

## Results

For *de novo* identification of distinct type I and type II IFN signatures, we applied a network meta-analysis-based workflow in which we combined the analyses of five bulk tissue RNA-seq datasets of IFN stimulation experiments from different healthy cellular environments. Further, we analyzed our signatures in three bulk tissue RNA-seq datasets and one PBMC scRNA-seq dataset to assess their translatability to data not used for discovery and to demonstrate that they can be applied in various disease contexts. To this end, we comprehensively compared our signatures with published IFN signatures. We examined the relevance of our signatures in two different disease contexts: Systemic Lupus Erythematosus (SLE) and cancer. To demonstrate their applicability across different technologies to measure gene expression, we assessed our signatures in microarray gene expression datasets from SLE and bulk tissue RNA-seq cancer datasets from TCGA and immune checkpoint inhibitor (ICI) trials. A scheme of the workflow and summary of our study is shown in Fig. 1A.

### Discovery of IFN response signatures through network meta-analysis

To obtain broadly applicable IFN gene modules that can inform on signals of both, IFN-I and IFN-II signaling separately, we leveraged five different in vitro/ex vivo bulk tissue RNA-seq datasets which study IFN stimulation in four different healthy tissue types (Table 1). Since we aimed at the discovery of IFN signatures that are applicable in different biological contexts, we included data for signature discovery that stem from diverse cell types or cellular contexts and that cover various experimental setups. Not all types of IFN stimuli that we assessed (IFN-a, IFN-b, and IFN-g) were uniformly applied in all five datasets. We aimed to discover IFN type-specific gene expression signatures by a holistic analysis of induced expression changes in all experiments. A traditional meta-analysis approach would obtain only direct estimates inferred in single studies, not taking indirect comparison of stimulations in different studies into account. Therefore, we decided to utilize an extension of traditional meta-analysis workflows, so-called network meta-analysis, to integrate gene-level results that we obtained from separate analyses of each of the five datasets (Fig. 1B).

First, we analyzed individual gene expression changes between IFN treatments and controls in each individual discovery dataset by performing DGE analysis on top common 5,000 genes with the highest variability (see Methods, Supp. Figure 2). The p-value distributions resulting from gene-wise tests in each dataset revealed an overabundance of low p-values in each experiment. This suggests a strong impact of IFN stimulation on many genes and the presence of sufficiently powered experiments to detect these [58], pointing to hundreds of differentially expressed genes for each type of IFN stimulation. These results suggest that the selected expression datasets are well suited for the discovery of specific IFN signatures in our analytical approach presented in the following paragraphs.

To estimate the expression changes on the gene level between different conditions, we applied network meta-analysis, one for each gene, on the outputs of all DGE analyses, i.e., log-fold change (logFC) and standard error SE for each treatment comparison: IFN-a vs. control, IFN-b vs. control, IFN-g vs. control, IFN-a vs. IFN-b, IFN-g vs. IFN-a, IFN-g vs. IFN-b (Methods, Fig. 1B). For each gene i, we obtained 4 × 4 matrices NM_i_ containing estimated FC values comparing different IFN treatments (IFN-a, IFN-b, and IFN-g) and controls.

To finally distill IFN subtype-specific upregulated genes from these results, we applied a two-tiered strategy by utilizing the NM_i_ matrix: First, we identified genes differentiating between IFN subtypes and second, we removed genes being upregulated in the control from the results of the treatment-versus-control comparisons in NM_i_.

We generated three distinct gene lists for comparing IFN treatment responses, namely IFN-a vs. IFN-b, IFN-a vs. IFN-g, and IFN-b vs. IFN-g. Applying stringent filtering criteria (|FC| > 2.5, FDR < 0.05), our results demonstrated 16 differentially expressed genes for IFN-a vs. IFN-b, 26 genes for IFN-a vs. IFN-g, and 79 genes for IFN-b vs. IFN-g. The limited number of distinctive genes between IFN-a and IFN-b suggests a high degree of biochemical similarity in their mode of action. The high number of differentially expressed genes between IFN-b and IFN-g suggests a considerable distinction regarding downstream transcriptional targets.

Next, we generated three gene lists for the comparison between each IFN subtype and the control. We specifically focused on genes with significantly high FC (FC > 3, FDR > 0.05) to concentrate on those exhibiting more substantial, treatment-dependent expression changes. Remarkably, IFN-g treatment revealed the least number of significant genes (*n* = 38), while IFN-b treatment yielded the highest number of treatment-affected gene sets (*n* = 111). This suggests that the gene expression changes induced by IFN-g were relatively limited with regard to the number of affected genes compared to other IFN treatments and IFN-b treatment had a more pronounced and widespread impact on the regulation of gene expression. This dual-filtering approach provided a comprehensive understanding of both IFN subtype-specific and treatment-specific gene expression patterns forming the basis for creating gene expression signatures for individual IFNs.

Combining these insights from our six comparative lists, we curated unique signatures for each individual IFN by exclusively considering the upregulated genes in specific comparisons, including subtype-level and treatment versus control analyses (Fig. 1B). To distill distinctive expression signals, we combined the sets of upregulated genes in comparisons involving a specific IFN with other IFNs forming the list of union of upregulated genes and intersected these results with those against the control. Notably, all IFN-a signature genes (*n* = 20) were present in the IFN-b signature, highlighting again how IFN-I subtypes might be biologically intertwined [59, 60]. Recognizing the challenge in isolating IFN-a- and IFN-b-specific signatures, we removed all IFN-a signature genes from the IFN-b signature. Consequently, there is no distinct IFN-a-specific signature, and the IFN-a signature is considered more broadly as IFN-I or IFN-a/b common signature. It is noteworthy that all individual genes in our IFN-g signature have been reported as IFN-g inducible genes in various contexts [61–65]. Finally, we derived our IFN signatures for IFN-a, IFN-b, and IFN-g (Table 2). During our assessment of our signatures, our three IFN signatures will be denoted as IFNa-Aybey, IFNb-Aybey and IFNg-Aybey, comprising 20, 40, 6 genes, respectively.

**Table 2:** Gene lists for our IFN signatures

IFN-a/ IFN_I_Aybey (*n* = 20)	CMPK2, DDX58, GMPR, HERC5, HERC6, HRASLS2, HSH2D, IFI27, IFI6, IFIT1, IFIT3, ISG15, LAMP3, MX1, MX2, OAS1, OAS2, OASL, RSAD2, USP18
IFN-b (*n* = 40)	BST2, BTC, C3AR1, CD7, CYP2J2, DDX60L, DHX58, DLL1, GCH1, IFI44, IFI44L, IFIH1, IFIT1B, IL22RA1, IL4I1, IRF7, ISG20, LGALS9, LMO2, MMP13, MYD88, NOD2, OAS3, PDGFRL, PLSCR1, PNPT1, PPM1K, RTP4, SAMD9, SIDT1, SLFN12L, SSTR2, STARD5, THEMIS2, TLR3, TMEM229B, TNFSF13B, TRANK1, TSPAN33, ZBP1
IFN-g/ IFN_II_Aybey (*n* = 6)	CD74, CXCL9, GBP2, ICAM1, IDO1, IRF1

## Selective IFN stimulation response of our IFN signatures in discovery and validation datasets

**Fig. 2:**
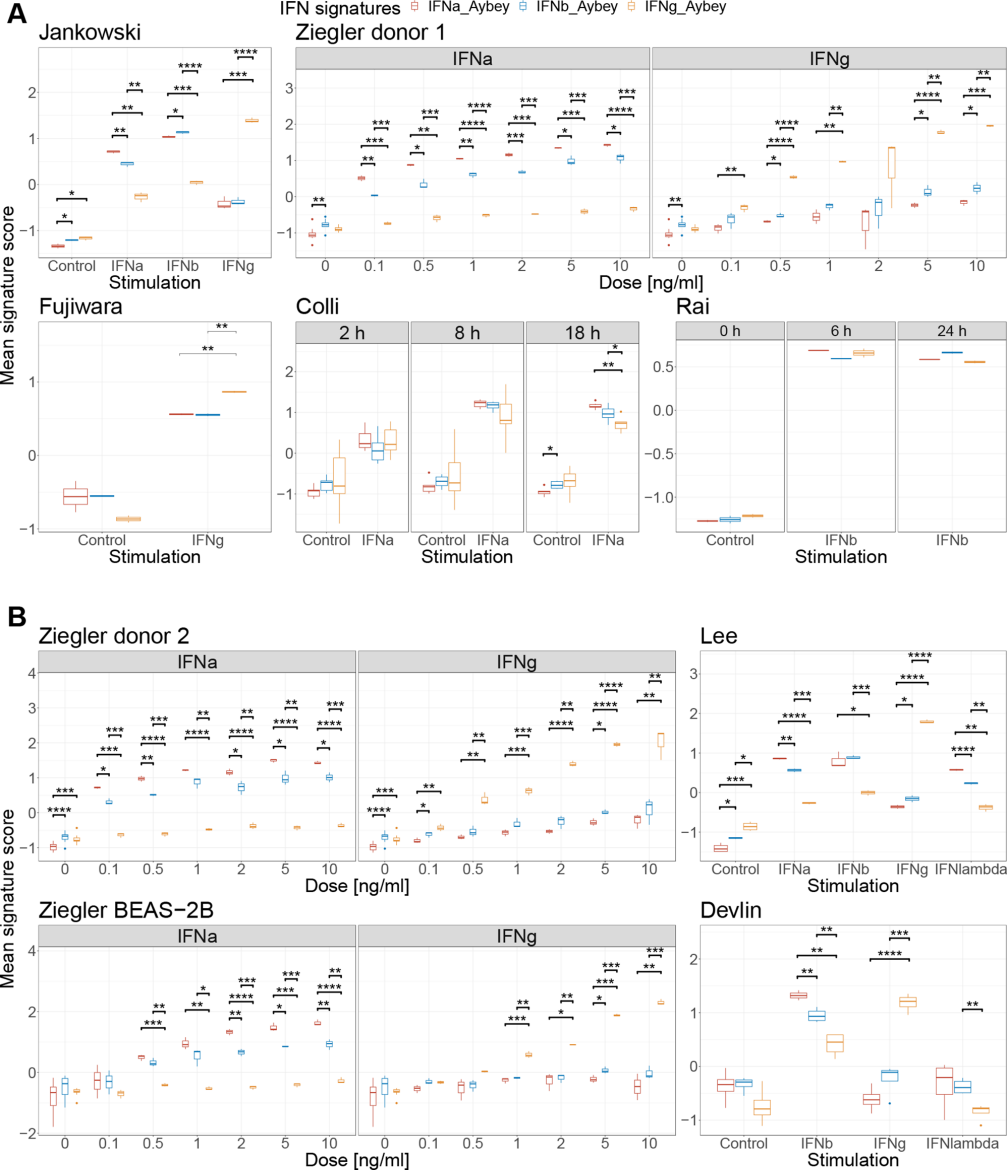
Discovery and validation of our IFN signatures. Mean signature scores for each signature are shown on the y-axis. Data points for IFNa-Aybey, IFNb-Aybey, and IFNg-Aybey signatures are colored differently in red, blue, orange, respectively. **(A)** Discovery datasets. In Ziegler dataset, on the x-axis, IFN concentrations are shown, and each box represents different stimulations while in other datasets different conditions are shown on the x-axis. Different boxes in Colli and Rai datasets represent different time points. **(B)** Validation datasets. In two Ziegler datasets, on the x-axis different IFN concentrations are shown while in the other two validation datasets, different conditions are shown on the y-axis. p-values comparing different IFN scores are shown. (* = *p* < 0.05, ** = *p* < 0.01, *** = *p* < 0.001, **** = *p* < 0.0001)

We examined whether different IFN signals can be distinguished using our IFN signatures by calculating mean signature expression scores for each signature, first in our discovery datasets (see Fig. 2A). We calculated the average changes in the signature score comparing each stimulation (d_mean_) against the unstimulated controls: The changes d_mean_ for IFNg-Aybey when samples were stimulated with IFNg were strong, and substantially higher compared to the changes observed for our IFNa-Aybey or IFNb-Aybey signatures upon IFN-g stimulation. Conversely, when we assessed IFN-a or IFN-b stimulations, the changes d_mean_ for IFNa-Aybey or IFNb-Aybey compared to IFNg-Aybey were substantially higher. These results were consistent across all discovery datasets for nearly all sampling time points after stimulation (see details for each dataset in Fig. 2), demonstrating the selectivity of our signatures for the given IFN subtype, either IFN-I or IFN-II.

Further, IFNa-Aybey exhibited a better differentiation from IFNg-Aybey than IFNb-Aybey did. For example, in discovery datasets with multiple IFN stimulations, the differences between d_mean_ between IFNg and IFNa scores and IFNg and IFNb scores ranged from 0 to 0.4 (Jankowski dataset) and 0 to 0.75 (Ziegler dataset) on the summed Z score scale. This indicates that our IFNa-Aybey signature is more distinct from IFNg-Aybey compared to IFNb-Aybey and therefore is better separating IFN-I and IFN-II signals. As expected, IFNa and IFNb signatures and stimulations were hardly bringing out significantly different patterns (d_mean_ ~ 0; Student’s t-test comparing IFNa and IFNb scores in each stimulation generally p-values > 0.05). This confirms that IFNa and IFNb downstream gene expression responses are closely related and hardly discernible by downstream gene expression programs. Based on these results we decided to use our IFNa-Aybey signature as our IFN-I signature and IFNg-Aybey as our IFN-II signature: analyses of these in discovery datasets consistently confirm their ability to separately assess IFN-I and IFN-II signals.

Next, we examined our IFN-I/II signatures across three diverse validation datasets, each representing distinct experimental contexts (Table 1; Fig. 2B). This additional validation aims to assess the applicability of our findings under varying experimental conditions. The investigation of the 2nd donor’s data and separate lung cell line BEAS-2B of the Ziegler dataset extended our discovery to a different donor of basal lung cells and as well to cell line contexts. IFN-I and IFN-II signals were separable showing increasing difference at increased doses (d_mean_ in the increasing range 0.4-2, and 0.6–2.8 for IFN-a and IFN-g stimulations, respectively; Student’s t-tests for comparisons of IFN-I and IFN-II scores in each dose level yields p-values < 0.05, respectively) validating the reliability of our signatures for studying IFN-I versus IFN-II response in a specific cellular model, here basal and epithelial lung cells.

Then, we assessed our signatures on the lung cell line Lee dataset [20] which covers a broad range of IFN stimulations (IFN-a, IFN-b, IFN-g and IFN-lambda). Similar to previous validation datasets, IFN-I and IFN-II signals can be separated with d_mean_ 2, 1.6, and 1.6 for IFN-a, IFN-b and IFN-g stimulations, respectively (Student’s t-test comparing IFN-I and IFN-II scores in each stimulation yields p-values < 0.05). Further, signals captured by our IFN signatures were more pronounced in IFN-a, IFN-b and IFN-g treatments compared with IFN-lambda treatment indicating the specificity of our IFN signatures to type I and II versus IFN-lambda. This demonstrated the validity of our signatures both in multiple IFN stimulation and additional lung cell line data.

The third validation dataset from Devlin et al. [28] posed a unique challenge for IFN signature assessment because it focuses on whole blood samples, a complex and heterogeneous cellular environment with multiple IFN stimulations (IFN-b, IFN-g and IFN-lambda). Our IFN signatures could still detect IFN-I and IFN-II signals reliably upon stimulation (d_mean_ 0.6 and 2.3 for IFN-b and IFN-g stimulations, Student’s t-test comparing IFN-I and IFN-II scores for each stimulation yields p-values < 0.05). In all validation datasets, we could detect distinct IFN-I and IFN-II signals. These findings suggest that our signatures are applicable in diverse complex biological samples, further supporting their utility in diverse experimental scenarios: under varying IFN doses, covering multiple IFN types, in complex and simple cellular environments.

For further analyses, we will focus on IFNa-Aybey as our IFN-I signature (IFN-I-Aybey) and IFNg-Aybey as our IFN-II signature (IFN-II-Aybey).

### Our IFN signatures are more coherent and differentiate IFN signals similarly or even better than published IFN signatures

**Fig. 3:**
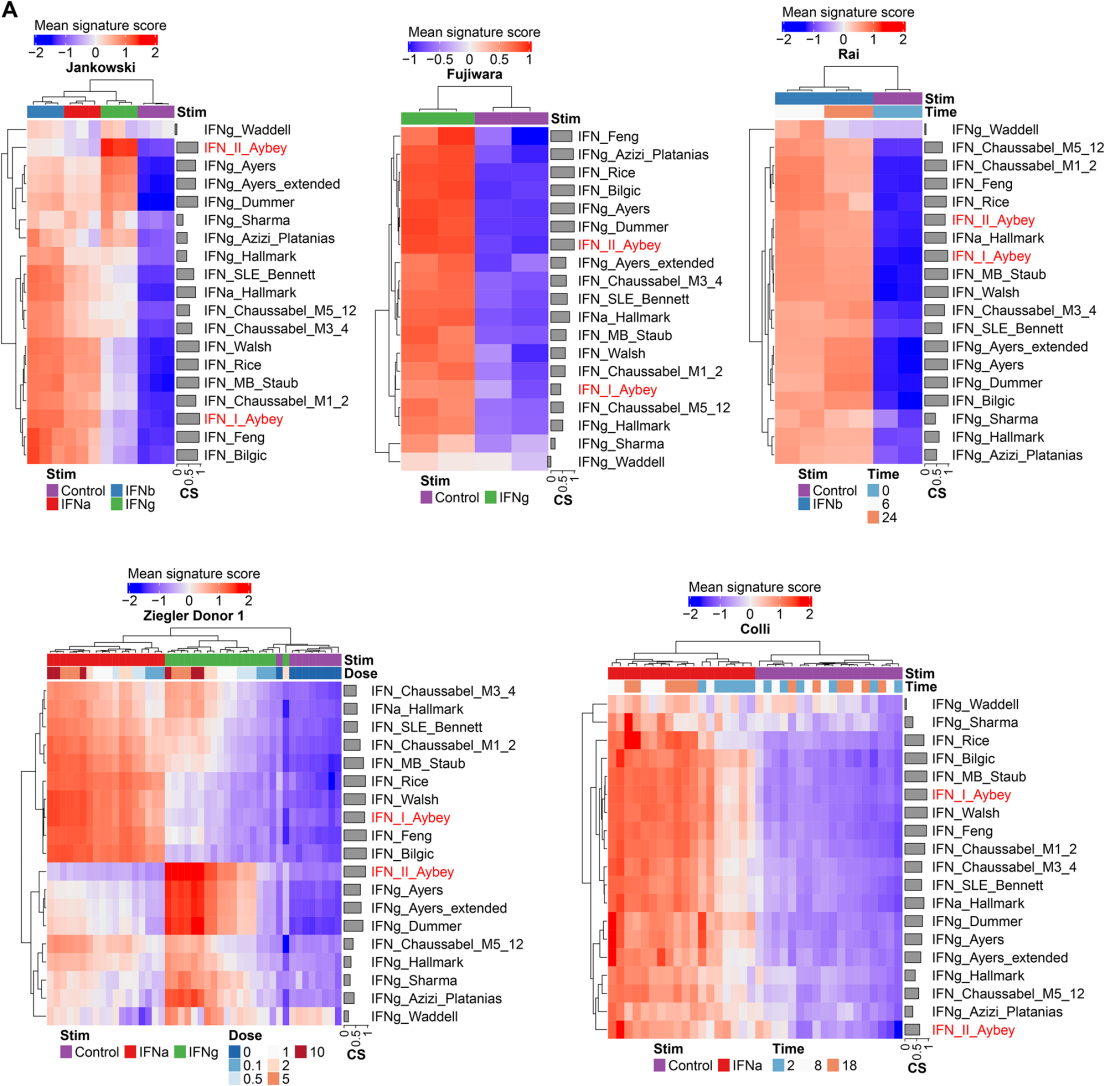

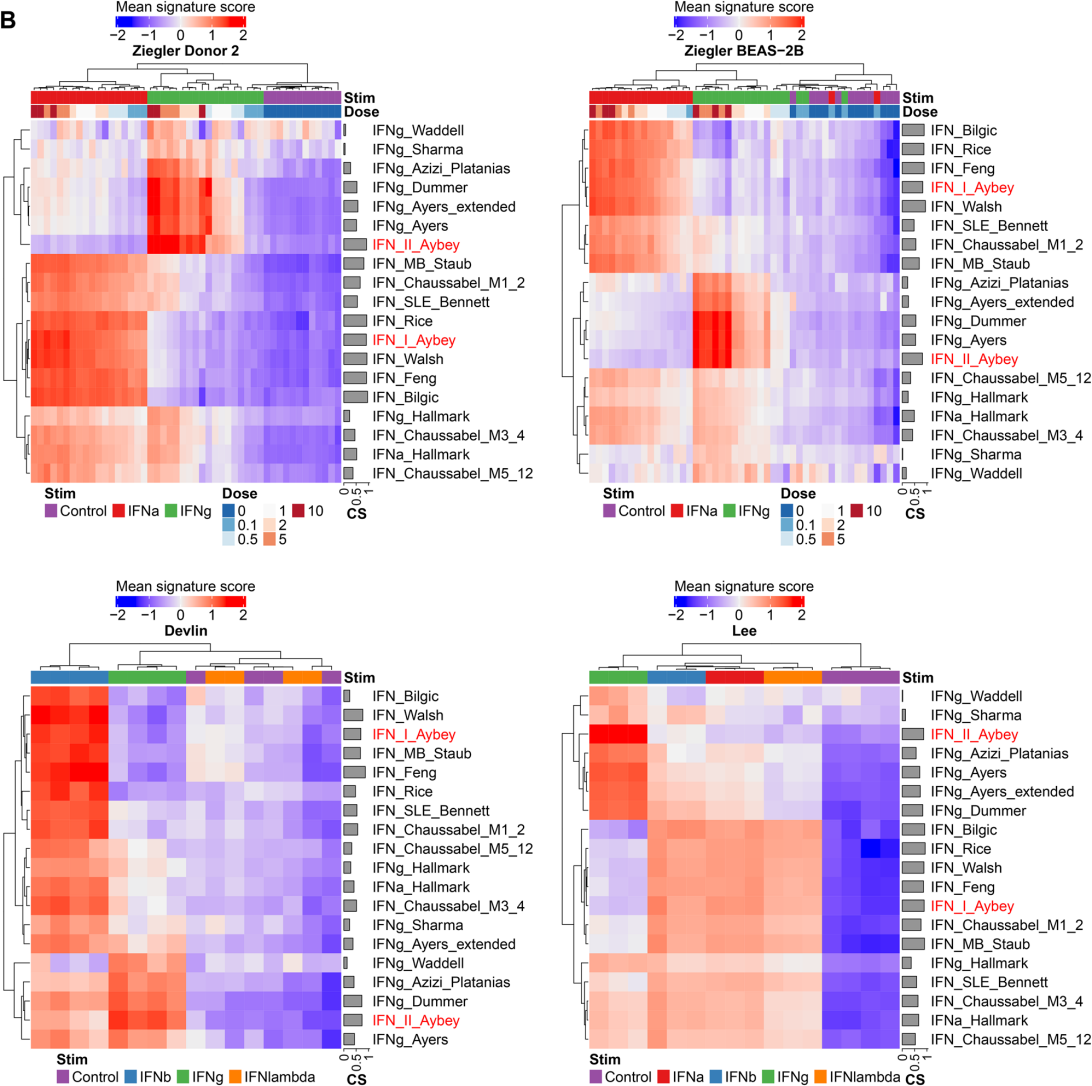
Comparison of our IFN signatures with published IFN signatures in the discovery and validation datasets. Mean signature score and coherence scores are shown for each signature separately in the discovery **(A)** and validation **(B)** datasets. Our IFN signatures are colored in red, others in black. Different experimental conditions such as time and IFN concentrations are shown on column annotations. Hierarchical clustering is applied column- and row-wise. Mean signature scores are shown from low (blue) to high (red)

To put our signatures into a broader perspective, we compared our IFN signatures with published IFN signatures: ten IFN-I and seven IFN-II signatures. We visualized the mean signature scores for each signature using clustered heatmaps of signature scores for the discovery and validation datasets (Fig. 3). Our IFN-I-Aybey signature clusters together with the majority of published IFN-I signatures. This group of similar IFN-I signatures yield higher scores in IFN-I stimulation experiments when compared with stimulations with other IFNs. There were other published IFN-I signatures which did not cluster together with our IFN-I-Aybey signature and the majority of other IFN-I signatures, such as IFNa-Hallmark [43], IFN-Chaussabel-M5-12 [8], and IFN-Chaussabel-M3-4 [8]. This finding suggests that they are not purely capturing IFN-I response. Indeed, these signatures were induced stronger or similar in other conditions when compared to IFN-I stimulation, and thus are less suited to demonstrate specific IFN-I response.

Our IFN-II-Aybey clustered together with most IFN-II signatures. This cluster of signatures could differentiate IFN-I and IFN-II signals more pronouncedly than other IFN-II signatures such as IFNg-Hallmark [43], IFNg-Waddell [51] and IFNg-Sharma [50] which could not discriminate IFN-II signal from other treatments.

These findings show that our signatures have similar characteristics to a considerably sized group of published IFN signatures that have sufficient discriminative power to discern IFN-I and IFN-II signals, while most published IFN signatures rather do not discriminate IFN-I and IFN-II signals.

To measure the relevance of the genes in each signature and assess the translatability of a signature to another dataset, we also calculated coherence scores (CS) for each signature [10] (Fig. 3). IFN-I-Aybey was among the most coherent IFN-I signatures and even had higher CS than some of those e.g., IFN-Rice [46], IFNa-Hallmark [43], IFN-SLE Bennet [7] and Chaussabel [8] signatures especially in the validation datasets. IFN-II-Aybey had the highest CS among all IFN-II signatures. Surprisingly, IFNg-Hallmark [43] had a rather low CS and yielded weak signals even in IFN-g stimulated samples, probably due to the large number of genes in the signature. Similarly, IFNg-Waddell [51] and IFNg-Sharma [50] yielded poor signal separation and low CS values. High coherence scores of our IFN signatures, especially of IFN-II-Aybey, compared to those of published signatures demonstrated the favorable representation of IFN signaling biology of our signatures and their promising translatability to further datasets. In summary, IFN Aybey signatures were significantly correlated with a subset of published IFN signatures that yield discriminatory signals for IFN-I and -II but showed similar or better signature coherence.

### Our IFN signatures can distinguish IFN-I/II signals in scRNA-seq data of immune cells

**Fig. 4:**
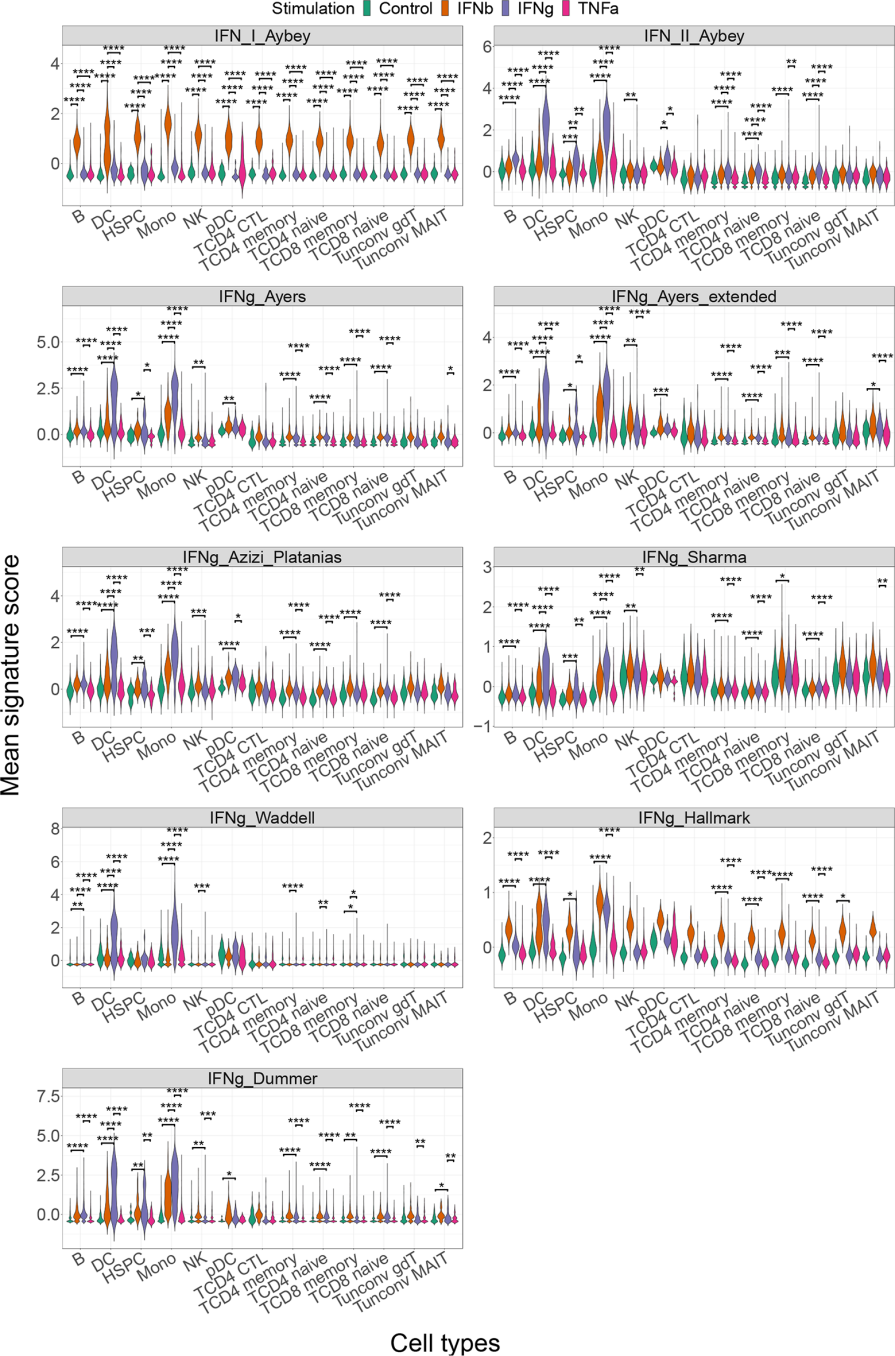
Our IFN-I and IFN-II signatures compared with published IFN-II signatures in IFN stimulation scRNA-seq data. For the evaluation of our IFN signatures and comparing those with published IFN-II signatures, GSE181897 scRNA-seq IFN stimulation dataset is used. Mean signature scores (y-axis) are calculated for each cell and shown for each signature (box) while cell type annotations defined by our random forest cell type classification method [37] are shown on the x-axis. The stimulations (IFN-b, IFN-g, and TNF-alpha) are shown in different colors. Each IFN-II score is pairwise compared in IFN-II treatment with other stimulations within each cell type using one-sided Student’s t-test and for IFN-I score comparison, IFN-I treatment is taken as reference. Bonferroni adjusted p values are displayed. (* = *p* < 0.05, ** = *p* < 0.01, *** = *p* < 0.001 **** = *p* < 0.0001)

Since the discovery and validation of IFN Aybey signatures mostly focused on bulk tissue RNA-seq datasets of isolated cell types, we assessed the utility of our IFN signatures to score gene expression of ex vivo IFN stimulated immune cells in a published scRNA-seq study (GSE181897) [34]. This external validation dataset comprises data from 64 healthy donors and covers multiple IFN stimulations (IFN-b and IFN-g). As a negative control, we included TNF-a stimulated cells from this dataset. We calculated mean signature scores for our signatures and projected them on a plot of cells that was based on a UMAP analysis in which only the data of genes from our previously published, robust immune cell type signatures were used [37] (Supp. Figure 3). IFN-I- and IFN-II-Aybey scores were exclusively high in IFN-b and IFN-g treated cell populations, respectively. Our IFN-I signature was highly expressed in all stimulated cell types while high scores for our IFN-II signature were limited mostly to myeloid and some other cells such as B cells. In addition to this confirmatory visualization of our signature scores demonstrating their applicability to single cell data, our signatures showed significantly higher mean signature scores in the respective IFN treatments in aforementioned cell types (one-way Student’s t-test p-adjusted < 0.05) compared to other conditions (Fig. 4).

Next, we asked if we could distinguish single IFN-I and IFN-II genes in this single cell IFN stimulation dataset. We performed a UMAP analysis on the gene level (not on the cell level as most often done in single cell analyses) based on all ISGs of either our signatures or of published IFN-I and IFN-II signatures. The UMAP plot separated IFN-II-Aybey genes to the exclusion of IFN-I-Aybey genes in the first UMAP dimension (Supp. Figure 4A). UMAP based on published gene sets did not show separation of different IFN types but rather that of common IFN-I/II genes (Supp. Figure 4B). This is evidence that our signature genes have the capability to distinguish IFN-I and IFN-II signals, even in a heterogenous single cell dataset.

### Our IFN-II signature detects cell type-specific IFN-II response better than published IFN-II signatures

We further examined how well our IFN-II-Aybey and other IFN-II signatures can detect cell type specific IFN-II responses. In GSE181897 single cell data [34], elevated IFN-II-Aybey scores were limited to certain cell type populations. As expected, myeloid cell populations showed the highest IFN-II response [51] (Fig. 4, Supp. Figure 3). Compared to other published IFN-II signatures, IFN-II-Aybey could detect (one-way Student’s t-test, p-adjusted < 0.05) IFN-II response in more cell types, especially in non-myeloid cell populations such as B cells, hematopoietic cells, and naïve T cell populations (Fig. 4). All other IFN-II signatures except the IFNg-Hallmark signature [43] yielded higher IFN-II scores predominantly in the myeloid cells after IFN-g treatment (one-way Student’s t-test p-adjusted < 0.05), but not in other cell types. Surprisingly, the IFNg-Hallmark signature [43] had higher scores after IFN-b treatment in all cell types, suggesting that this signature comprises a mix of IFN-I/II genes instead of being specific for IFN-g. Further, some signatures such as IFNg-Sharma [50] showed cell type-specificity rather than IFN-II specificity: it yields overly high scores in T and NK cell populations independent of IFN stimulation. Some signatures such as IFNg-Ayers [6] and IFNg-Azizi-Platanias [48, 49] had higher IFN-II score after IFN-b treatment in T and NK cell populations. Additionally, by analyzing an independently published temporal IFN-g stimulation scRNA-seq experiment [36], we confirmed the IFN-g activation of non-myeloid cells such as B cells and naïve T cell populations using IFN-II-Aybey signature (one-way Student’s t-test comparing IFN-II-Aybey score in IFN-g against control at 1–6 h, p-adjusted < 0.05) (Supp. Figure 5). In summary, our IFN Aybey signatures demonstrated a superior capability to characterize IFN responses in this single cell dataset: in particular, they were better capable to characterize IFN-I versus IFN-II signals in different cell types than other published signatures.

### Our IFN-I signature is highly coherent and is associated with disease severity in SLE

We examined the relevance of our signatures in the context of the autoimmune disease Systemic Lupus Erythematosus (SLE). In SLE, elevated IFN-I signaling is of particular importance, and has been described to result in an association of IFN-I signatures and SLE disease activity index (SLEDAI) scores, a measure of disease severity [15]. To showcase the applicability of our signatures in three independent microarray SLE patient cohorts, we assessed CSs on these datasets and calculated Spearman correlation (SC) coefficients between IFN signature scores and baseline SLEDAI scores in SLE patients of these cohorts (Table 3). Our signatures had high (IFN-I-Aybey 0.55–0.71) or moderate (IFN-II-Aybey ~ 0.15) CSs in all three datasets, suggesting that there is a significant degree of coordinated up- or down-regulation of signature genes across patients which means that signature scores are meaningful representations of IFN-I/-II module regulation across patients.

Several IFN-I signatures were strongly correlating with SLEDAI scores. IFN-I-Aybey was among the signatures with best correlation to SLEDAI: exhibiting higher or similar SC (0.2–0.5) than other IFN signatures, even those that were derived from SLE datasets e.g., Bennet [7], and Chaussabel [8]. This demonstrates the relevance of our IFN-I signature in the SLE context. Surprisingly, all IFN-II signatures except the IFNg-Hallmark signature [43] (which comprises a high number of ~ 200 genes and most likely is not a pure IFNg signature, see results in Figs. 3 and 4) had low SC (< 0.2) with SLEDAI. IFN-II-Aybey also had low SC which indicates a lack of association of IFN-II signaling to disease severity in SLE and is in line with previous research around IFN-I/II and their role in SLE [7, 8]. These results demonstrate the relevance of our IFN-I-Aybey signature in SLE and confirmed the weak association of IFN-II signatures with SLE disease severity.

**Table 3:** Spearman correlation between signature scores and SLEDAI along coherence scores for signatures in SLE datasets

IFN signature	Spearman correlation			Coherence score		
	GSE121239	GSE49454	GSE65391	GSE121239	GSE49454	GSE65391
IFN_SLE_Bennett (26)	0.34	0.2	0.48	0.51	0.44	0.45
IFN_Chaussabel_M3_4 (59)	0.34	0.14	0.5	0.52	0.5	0.54
IFN_Chaussabel_M5_12 (58)	0.32	0.21	0.47	0.35	0.36	0.36
IFN_I_Aybey (20)	0.32	0.19	0.46	0.71	0.58	0.55
IFN_Walsh (6)	0.32	0.16	0.45	0.93	0.94	0.91
IFN_Chaussabel_M1_2 (32)	0.32	0.11	0.45	0.79	0.62	0.64
IFNa_Hallmark (97)	0.31	0.11	0.46	0.35	0.31	0.31
IFN_Rice (4)	0.3	0.17	0.43	0.92	0.8	0.62
IFN_Bilgic (3)	0.31	0.16	0.42	0.76	0.39	0.13
IFN_Feng (5)	0.3	0.11	0.44	0.92	0.78	0.72
IFN_MB_Staub (10)	0.31	0.08	0.42	0.78	0.64	0.63
IFNg_Hallmark (200)	0.25	0.05	0.43	0.13	0.14	0.15
IFNg_Dummer (5)	0.09	-0.12	0.21	0.29	0.06	0.11
IFNg_Waddell (10)	-0.02	0.11	0.12	0.12	0.07	0.09
IFNg_Azizi_Platanias (11)	-0.01	-0.14	0.15	0.07	0.04	0.07
IFN_II_Aybey (6)	-0.12	-0.09	0.15	0.16	0.13	0.14
IFNg_Ayers (6)	0.06	-0.19	0.09	0.22	0.17	0.15
IFNg_Ayers_extended (10)	0.01	-0.21	0.06	0.25	0.13	0.12
IFNg_Sharma (25)	-0.11	-0.27	0.03	0.17	0.18	0.18

The number of genes for each IFN signature is shown in brackets. IFN signatures are ordered by the ranking based on correlation coefficients in three SLE datasets

### Our IFN signatures can disentangle type I versus type II IFN signals in cancer gene expression datasets

**Fig. 5:**
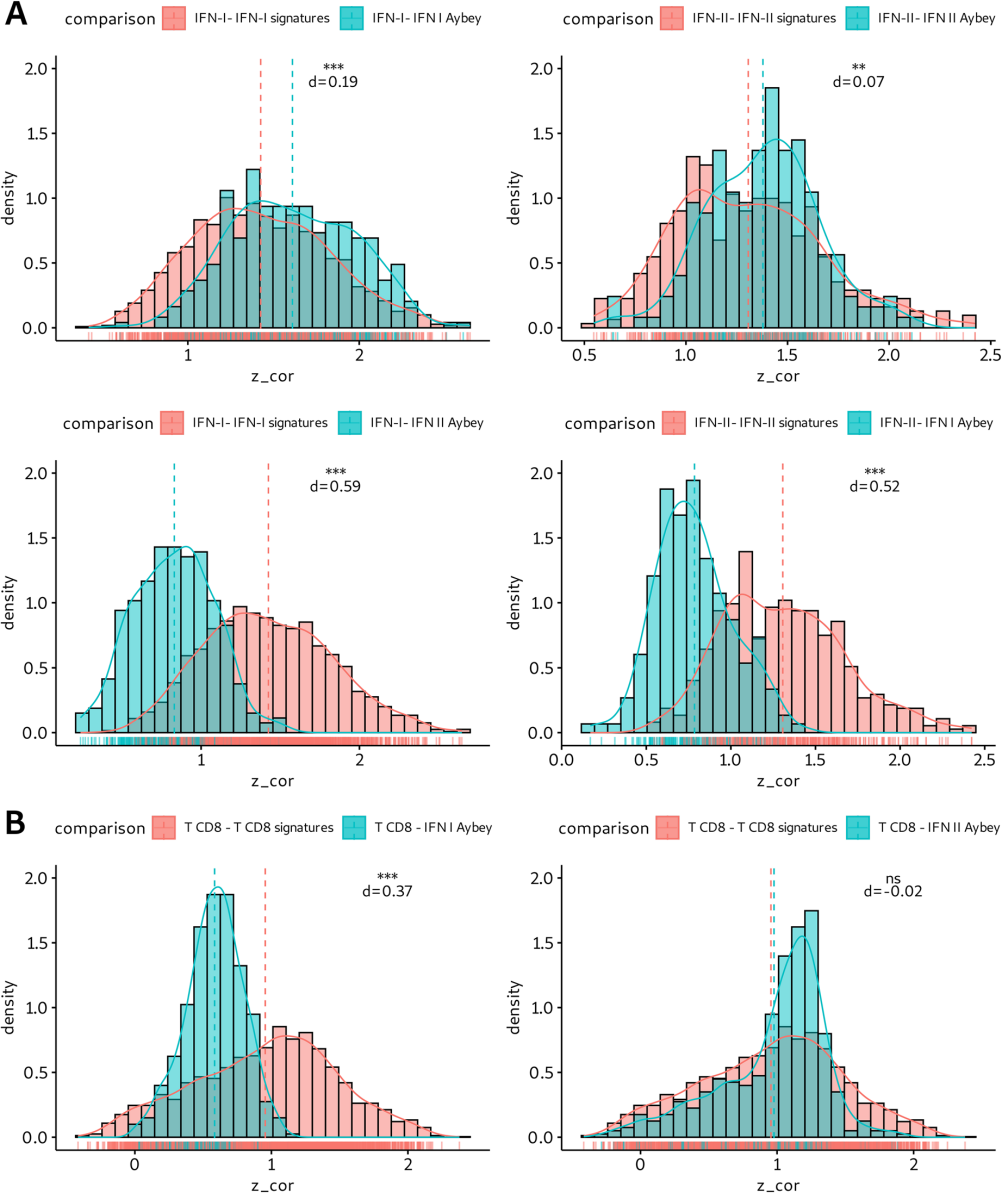

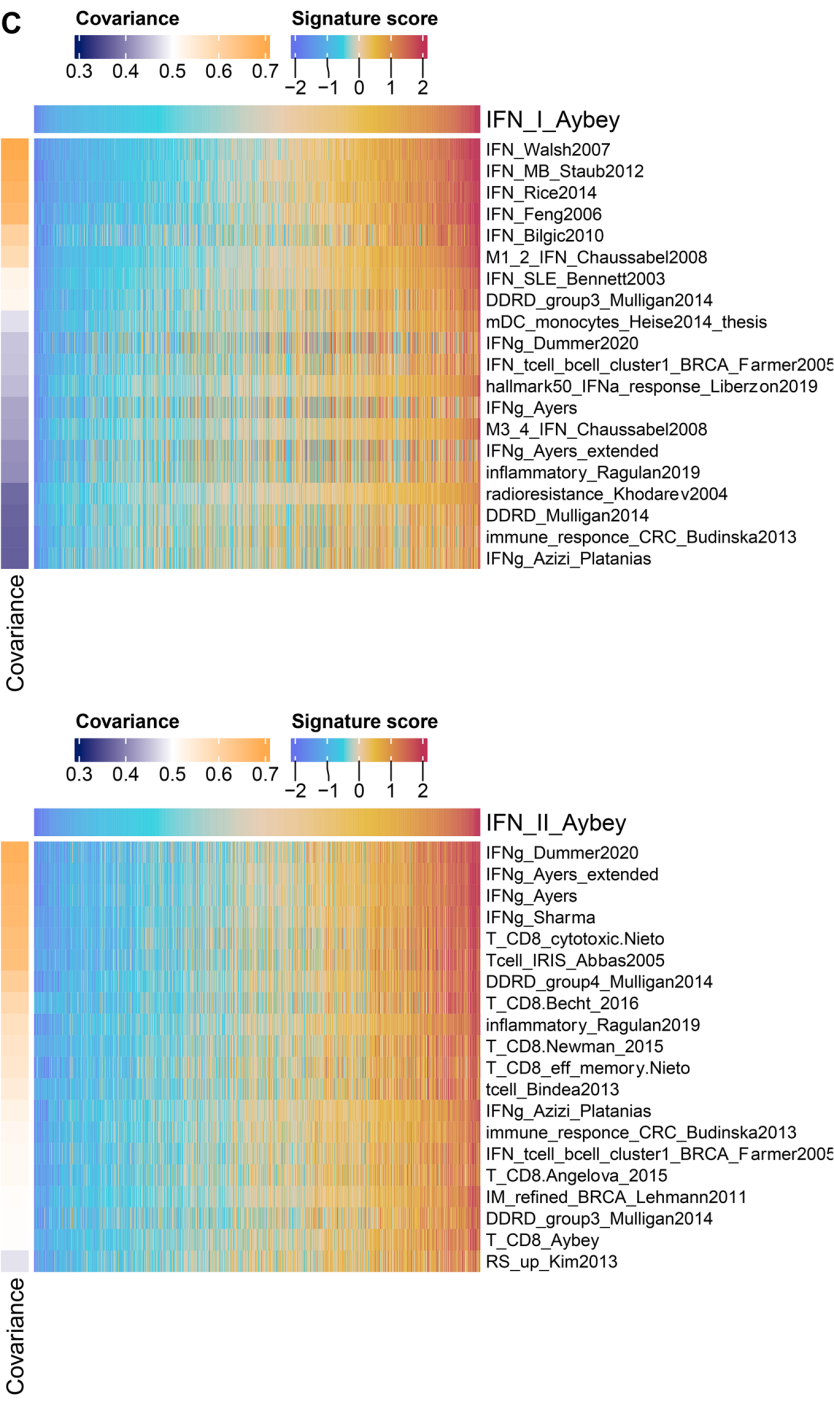
**(A-B)** Correlation histograms between ours and published IFN signatures or CD8^+^ T cells in TCGA cohorts. Mean signature scores for each signature are calculated in each TCGA cohort (*n* = 32) separately. Within each cohort Pearson correlation coefficients are calculated between our IFN signatures and all other published signatures for IFN-I, IFN-II or CD8^+^ T cells. Correlation values are z-transformed (Fisher transformation) and shown on x-axis while y-axis represents the density of the distributions. Each color shows different comparisons e.g., IFN-I against IFN-I or IFN-I against IFN-I-Aybey. Two-sided Student’s t-test is used to compare the similarity between the distribution of the histograms, i.e., the means of the distributions. (ns = non-significant (p-value > 0.05); *** = significant (p-value < 0.001). The differences between the means of the histograms are denoted as d. **(C)** 20 signatures with highest covariance to our IFN signatures in TCGA BRCA cohort. A similar analysis from Kreis et al. is performed using the signatures from Kreis et al. and additional published CD8^+^ T cell and IFN-II signatures [16]. Mean signature scores are calculated only for coherent signatures (coherence score > 0.2). Covariance between each of our IFN signatures and coherent signatures are calculated and the highest 20 signatures are displayed in rows. Mean signature scores are shown from low (blue) to high (red). Covariance values are shown from low (dark blue) to high (orange)

We asked if we could separate different IFN signals using our IFN signatures in cancer bulk tissue RNA-seq datasets. We focused our analysis on the TCGA expression data of all 32 cohorts. For each cohort, we calculated the Pearson correlation between the mean per-sample signature scores of our IFN signatures and all published IFN signatures. We transformed the correlation coefficients by Fisher’s z-transform into Z scores that should follow a normal distribution and plotted these in histograms (Fig. 5A). The mean correlation Z score of comparisons of IFN-I-Aybey with other published IFN-I signatures was slightly but significantly higher to that of the correlation Z scores that stem from pairwise comparisons within the set of published IFN-I signatures (d_mean_= 0.19, two-sided Student’s t-test, *p* < 0.001). The same trend was observed when comparing IFN-II-Aybey and other published IFN-II signatures and contrast this pairwise comparisons within the group of published IFN-II signatures (d_mean_= 0.07, two-sided Student’s t-test, *p* < 0.01). This demonstrates that our IFN signatures are not only highly correlated with published IFN signatures; they stand out in their ability to match the scores of all the published ones.

In contrast, the mean correlation Z score of IFN-II-Aybey with other IFN-I signatures compared with pairwise comparisons from other IFN-I signatures was strongly and significantly lower (d_mean_= 0.59, two-sided Student’s t-test, *p* < 0.001) (Fig. 5A). This was observed analogously for IFN-I-Aybey when compared with IFN-II signatures (d_mean_= 0.52, two-sided Student’s t-test, *p* < 0.001). We summarize that our IFN-I and IFN-II signatures can yield distinct signals for IFN-I/-II stimulations and their ability to yield separate signals is higher than that for other published IFN-I or IFN-II signatures.

### Our IFN-II signature correlates with CD8^+^ T cell infiltration into tumor tissue and with response to immune checkpoint inhibitor therapy

Further, we analyzed the relationship between CD8^+^ T cell infiltration and IFN-II response using the correlation Z scores between our IFN signatures and published CD8^+^ T cell signatures. The mean correlation Z scores resulting from pairwise comparisons among different CD8^+^ T cell signatures surpassed the mean correlation Z score between IFN-I-Aybey and CD8^+^ T cell signatures (d_mean_= 0.37, two-sided Student’s t-test, *p* < 0.001) (Fig. 5B). Conversely, the mean correlation Z score of IFN-II-Aybey with CD8^+^ T cell signatures was notably lower and even significantly showed the same distribution (d_mean_= 0.02, two-sided Student’s t-test, *p* > 0.05). These results demonstrate a close relationship between CD8^+^ T cell infiltration and IFN-II response and suggest a weaker influence of IFN-I signaling.

In addition, we carried out a RosettaSX gene expression signature analysis as proposed by Kreis et al. on an exemplary TCGA BRCA cohort [16]. We analyzed the patterns of highly coherent signatures together with our IFN signatures, coherent signatures of various functions as used by Kreis et al., and further published IFN-I, IFN-II and CD8^+^ T cell signatures: we sorted the signatures by their covariance to our IFN signatures across patients (Fig. 5C, Supp. Figure 6) [16]. As expected, other IFN-I signatures showed similar expression patterns to IFN-I-Aybey. Surprisingly, some IFN-II signatures such as IFNg-Dummer [9] and IFNg-Ayers [6] showed high covariance when compared with IFN-I-Aybey (covariance > 0.3). Our IFN-II-Aybey signature was among the IFN-II signatures with the least covariance to our IFN-I-Aybey. This finding confirms the separability between our IFN signatures in cancer gene expression data, here with the TCGA BRCA dataset as an example.

When ranking other signatures for similarity to IFN-II-Aybey in these data, IFN-II and CD8^+^ T cell signatures were among the most similar expressed signatures. Among 20 signatures with highest covariance to IFN-II-Aybey no IFN-I signature was present. Interestingly, IFNg-Hallmark [43] signature was the signature with least covariance to IFN-II-Aybey in comparison to other IFN-II signatures. Overall, in TCGA cohorts IFN-II-Aybey was well separable from published IFN-I signatures, highly correlated and related to published CD8^+^ T cell signatures.

Finally, we extended our analysis into the context of cancer treatment effects. Recently higher IFN-II scores have been reported in responders before ICI therapy [6, 17–19]. Using our IFN signatures we tested this hypothesis in three independent ICI trial cohorts of three different cancer types: bladder cancer [33], melanoma [31], and gastric cancer [30].

**Fig. 6:**
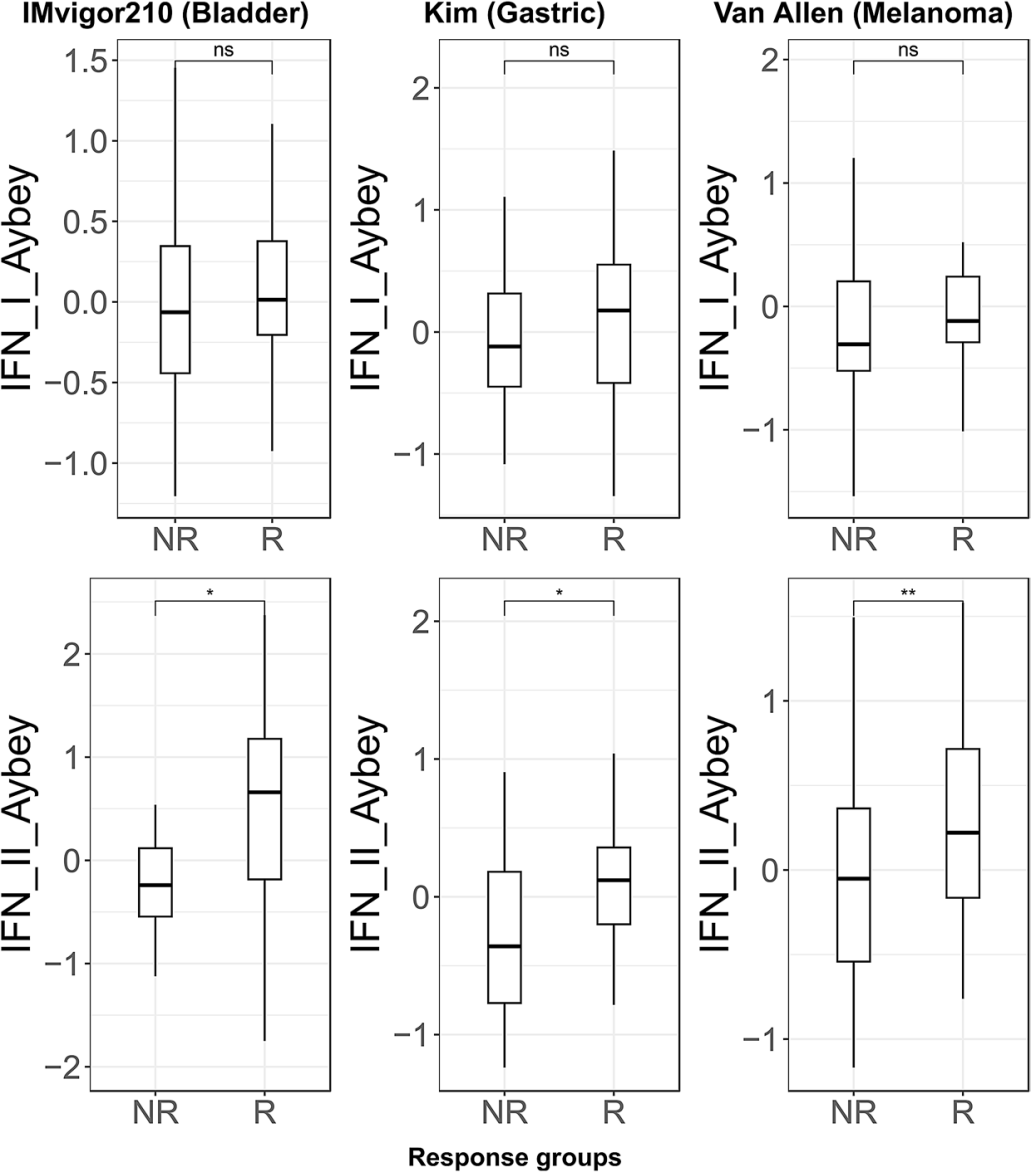
Boxplots of IFN-I and IFN-II Aybey signature scores in ICB therapy responders versus non-responders. Three bulk tissue RNA-seq datasets of three different cancer types are used: gastric cancer (30), melanoma (31) and bladder cancer- IMvigor210 (33) are used for the analysis. Mean signature scores for each signature (y-axis) are calculated in each cohort separately. Responders (R) and non-responders (NR) are shown on the x-axis. Two-sided Student’s t-test is used to compare differences in the mean signature scores between R and NR. (ns = non-significant (p-value > 0.05); * = *p* < 0.05, ** = *p* < 0.01* and ** = significant (p-value < 0.05 or 0.01, respectively)

For each cancer sample (collected at treatment baseline), we calculated mean signature scores for our signatures. IFN-II-Aybey scores were significantly higher (two-sided Student’s t-test, *p* < 0.01) in the responders whereas IFN-I-Aybey scores were same or not significantly higher (two-sided Student’s t-test, *p* > 0.05) (Fig. 6). Further, we investigated the predictive power of IFN-II-Aybey compared to other IFN signatures for ICI response using a logistic regression model in ICI-treated cohorts. Based on the prediction probabilities, we conducted receiver operating characteristic (ROC) analysis. ROC analysis demonstrated the consistent predictive performance of IFN-II-Aybey signature in ICI-treated cohorts, with an area under the ROC curve (AUC) of approximately 0.7 across three clinical studies (Table 4). It ranked among the top-performing IFN-related markers, comparable to IFNg-Dummer and IFNg-Azizi-Platanias, and following the reference IFNg-Ayers signature. These findings support the potential clinical utility of the IFN-II-Aybey signature as a biomarker for ICI responsiveness and highlight its applicability in diverse immuno-oncology settings. At least for these three independent cohorts, our IFN-II-Aybey signature recapitulated previously published reports showing that IFN-II response and signaling at baseline were predictive of better clinical outcome patients and we could demonstrate the lack of association of IFN-I signaling in baseline response.

**Table 4:** ROC analysis of published IFN signatures on three ICI therapy datasets

Signature	AUC IMvigor210	AUC Kim	AUC Van Allen
IFNg_Ayers	0.64	0.8	0.7
IFNg_Ayers_extended	0.63	0.77	0.72
IFNg_Dummer	0.67	0.79	0.66
IFNg_Azizi_Platanias	0.64	0.78	0.69
IFN_II_Aybey	0.62	0.77	0.7
IFNg_Waddell	0.64	0.72	0.72
IFNg_Sharma	0.6	0.74	0.73
IFN_Chaussabel_M3_4	0.57	0.78	0.66
IFNa_Hallmark	0.57	0.75	0.65
IFNg_Hallmark	0.57	0.75	0.66
IFN_Chaussabel_M5_12	0.57	0.72	0.62
IFN_MB_Staub	0.58	0.66	0.62
IFN_SLE_Bennett	0.58	0.69	0.6
IFN_Rice	0.55	0.7	0.61
IFN_Chaussabel_M1_2	0.58	0.68	0.57
IFN_I_Aybey	0.58	0.6	0.59
IFN_Feng	0.55	0.65	0.55
IFN_Walsh	0.58	0.62	0.53
IFN_Bilgic	0.51	0.63	0.53

Area under the ROC curve (AUC) values for each signature are represented and signatures are sorted by overall mean AUC values across three studies

## Discussion

Most published IFN signatures have been obtained based on analyses of only single gene expression datasets. Only some have been obtained by analyses of multiple datasets, then being restricted to a specific disease or tissue context. These signatures have not undergone a comprehensive assessment of IFN responses on the single cell level. We show that most of the published IFN signatures hardly distinguish between type I and type II IFN responses: they are mostly general IFN signatures or rather IFN-I signatures. In this study, we intended to include gene expression datasets obtained from different tissues and experimental designs (time courses, concentration ranges and types of IFN stimulation) to obtain signatures that are more specific about the type of IFN response that they can monitor, or more general regarding their use in different disease contexts. To our knowledge, no other IFN gene signature set has been published yet that can differentiate IFN-I and IFN-II response in multiple IFN stimulation gene expression data of healthy cellular environments. Therefore, we believe our IFN-I and IFN-II signatures can be helpful tools to understand and further characterize IFN-I and -II response in healthy cells of different types which is a prerequisite to a better understanding of the actions of immune cells in diseases.

We discovered our signatures using multiple bulk tissue RNA-seq datasets. We tested the applicability of our signatures in diverse gene expression datasets (bulk tissue RNA-seq, microarray and scRNA-seq), multiple cell types and various disease contexts. In all cases our signatures could separate IFN-I and IFN-II specific signals well and yield high signature coherence. Therefore, we are confident that our signatures are valid tools to interrogate IFN response for gene expression data produced by different technology platforms and biological specimen from bulk tissue to single cells.

In disease contexts, especially when complex tissues are profiled, IFN-I and- II signaling is often difficult to entangle by gene expression profiling, but even in heterogenous cancer cohorts from TCGA we could differentiate our IFN-I signatures from IFN-II signatures well and could show an association of our IFN-II signature to published CD8^+^ T cell signatures (Fig. 5) clustering to the exclusion of IFN-I signatures. Our signatures were disease-relevant in SLE where our IFN-I-Aybey was among the best signatures regarding its correlation with SLEDAI scores (Table 3). Our IFN-II-Aybey had overall the highest coherence among other IFN-II signatures. In cancer gene expression studies, scores of our IFN-II-Aybey signature were associated with response to ICI treatment: we could observe higher IFN-II signature signals in ICI responders in pre-treatment samples (Fig. 6). Our findings of the role of IFN-I and IFN-II signatures in the context of SLE disease severity and ICI response of cancer patients confirms previous findings: however, the availability of a set of homogeneously derived and distinguishable IFN-I and IFN-II signatures with relevance across diverse disease contexts is unprecedented and suggest broad utility of our signature set.

We realized that for IFN-I responses a fine-grained separation of IFN-a versus IFN-b stimulation was not possible: all our IFN-a stimulated genes were also present in our IFN-b signature. Therefore, we used the common genes as our IFN-I signature. The data available to us has not been sufficient to identify genes that are specifically regulated in IFN-a versus IFN-b stimulation. It is not surprising that the canonical signaling of both IFNs converges at the same set of transcriptional regulators [2]. Our aim to differentiate IFN-a and IFN-b response using our meta-analysis approach could not be fulfilled, even though type I and II response could be differentiated. This suggests that IFN-a and IFN-b are indeed a largely redundant signaling component in the experimental contexts that we investigated. This underlines the central importance of type I IFN response in innate immunity.

Since there was no extensive comparison and evaluation of published IFN signatures in the previous publications, we also included other published IFN signatures in our analysis. Our signatures mostly showed higher coherence and better signal-to-noise ratios than published signatures, and covered IFN-II responses of more cell types. IFN-II-Aybey could detect more fine-grained IFN response of hemopoietic stem cells, subpopulations of B cells and naïve T cells (Fig. 4). Compared to other published IFN signatures, we could clearly separate IFN-I and IFN-II genes in UMAP analyses of our IFN-I or -II signature genes in an independent dataset (Supp. Figure 4). Surprisingly, we found that some published signatures such as IFNg-Sharma were cell type-specific in their capability to show IFN-g response while others such as IFNg-Hallmark clearly showed signs of a mixed IFN-I/II signal, i.e., high scores in both IFN-I and IFN-II treatments (Figs. 3 and 4). We suggest using published IFN signatures with caution when used in contexts for which they had not been designed: their cell type or treatment specificities should be validated before their application in a new disease or tissue context.

One limitation of our study is that our gene signature scores are derived from Z-score normalization, making the scorings cohort-dependent. This may affect the reproducibility and applicability of these signatures in prospective studies or across different experimental methodologies if characteristics of new patient cohorts deviate significantly from our investigated cohorts. Future studies could address this limitation by employing co-normalization techniques, where new samples are normalized alongside our published datasets. The development of single-sample classification schemes for specific applications will be the target of future studies. Such approaches could help mitigate platform-specific biases and enhance the robustness of the gene signatures across diverse application areas.

In summary, we obtained coherent, disease-relevant, and broadly applicable IFN-I and IFN-II signatures which can be utilized in multiple cellular environments, disease contexts and on gene expression data produced by different methodologies. Our IFN-II-Aybey signature showed superior IFN-II signal detection in various cell types which was not possible with previously published IFN-II signatures. In the context of diseases like cancer and SLE, we could recapitulate previous findings and phenomena regarding the relation of IFN signaling to disease parameters or immunotherapy response. For emerging single cell datasets, we argue that our IFN signature set will be a useful tool to examine IFN response in a more fine-grained way than previously possible: providing highly relevant (as demonstrated by strong coherence) per-sample scores and proven IFN-I versus IFN-II signal specificity in the context of analyses of complex tissues or single cells from healthy and diseased tissue.

## Electronic supplementary material

Below is the link to the electronic supplementary material.

Supplementary Material 1

## Data Availability

All publicly available datasets used in this study have been listed in the methods section. The scripts to perform the analyses of this study is deposited on https://github.com/ba306/Interferon-signature-discovery-analysis.

## Abbreviations

CS: Coherence Score
DGE: Differential Gene Expression
DEGs: Differentially Expressed Genes
FC: Fold Change
FPKM: Fragments per Kilobase of Transcript per Million Mapped Reads
ICI: Immune Checkpoint Inhibitor
IFN: Interferon
IFN-beta: IFN-b
IFN-gamma: IFN-g
IFN-I: Type-I IFN
IFN-II: Type-II IFN
IFN-III: Type-III IFN
ISG: IFN-Stimulated Genes
SC: Spearman Correlation
SLE: Systemic Lupus Erythematosus
SLEDAI: SLE Disease Activity Index
TNF-alpha: TNF-a
TPM: Transcripts per Kilobase Million

## References

[1] Mesev EV, LeDesma RA, Ploss A. Decoding type I and III interferon signalling during viral infection. Nat Microbiol. 2019;4(6):914–24. 30936491 10.1038/s41564-019-0421-x PMC6554024

[2] Chow KT, Gale M. Jr. SnapShot: interferon signaling. Cell. 2015;163(7):1808–e1. 26687364 10.1016/j.cell.2015.12.008

[3] Garcin G, Bordat Y, Chuchana P, Monneron D, Law HK, Piehler J, et al. Differential activity of type I interferon subtypes for dendritic cell differentiation. PLoS ONE. 2013;8(3):e58465. 23472200 10.1371/journal.pone.0058465 PMC3589429

[4] James CM, Abdad MY, Mansfield JP, Jacobsen HK, Vind AR, Stumbles PA, et al. Differential activities of alpha/beta IFN subtypes against influenza virus in vivo and enhancement of specific immune responses in DNA vaccinated mice expressing haemagglutinin and nucleoprotein. Vaccine. 2007;25(10):1856–67. 17240000 10.1016/j.vaccine.2006.10.038

[5] Schreiber G. The molecular basis for differential type I interferon signaling. J Biol Chem. 2017;292(18):7285–94. 28289098 10.1074/jbc.R116.774562 PMC5418031

[6] Ayers M, Lunceford J, Nebozhyn M, Murphy E, Loboda A, Kaufman DR, et al. IFN-gamma-related mRNA profile predicts clinical response to PD-1 Blockade. J Clin Invest. 2017;127(8):2930–40. 28650338 10.1172/JCI91190 PMC5531419

[7] Bennett L, Palucka AK, Arce E, Cantrell V, Borvak J, Banchereau J, et al. Interferon and granulopoiesis signatures in systemic lupus erythematosus blood. J Exp Med. 2003;197(6):711–23. 12642603 10.1084/jem.20021553 PMC2193846

[8] Chaussabel D, Quinn C, Shen J, Patel P, Glaser C, Baldwin N, et al. A modular analysis framework for blood genomics studies: application to systemic lupus erythematosus. Immunity. 2008;29(1):150–64. 18631455 10.1016/j.immuni.2008.05.012 PMC2727981

[9] Dummer R, Brase JC, Garrett J, Campbell CD, Gasal E, Squires M, et al. Adjuvant Dabrafenib plus Trametinib versus placebo in patients with resected, BRAF(V600)-mutant, stage III melanoma (COMBI-AD): exploratory biomarker analyses from a randomised, phase 3 trial. Lancet Oncol. 2020;21(3):358–72. 32007138 10.1016/S1470-2045(20)30062-0

[10] Staub E. An interferon response gene expression signature is activated in a subset of Medulloblastomas. Transl Oncol. 2012;5(4):297–304. 22937182 10.1593/tlo.12214 PMC3431040

[11] Jabs WJ, Hennig C, Zawatzky R, Kirchner H. Failure to detect antiviral activity in serum and plasma of healthy individuals displaying high activity in ELISA for IFN-alpha and IFN-beta. J Interferon Cytokine Res. 1999;19(5):463–9. 10386858 10.1089/107999099313901

[12] El-Sherbiny YM, Psarras A, Md Yusof MY, Hensor EMA, Tooze R, Doody G, et al. A novel two-score system for interferon status segregates autoimmune diseases and correlates with clinical features. Sci Rep. 2018;8(1):5793. 29643425 10.1038/s41598-018-24198-1 PMC5895784

[13] Pinto EF, Andrade C, Interferon-Related Depression. A primer on mechanisms, treatment, and prevention of a common clinical problem. Curr Neuropharmacol. 2016;14(7):743–8. 26733280 10.2174/1570159X14666160106155129 PMC5050402

[14] Bengtsson AA, Ronnblom L. Role of interferons in SLE. Best Pract Res Clin Rheumatol. 2017;31(3):415–28. 29224681 10.1016/j.berh.2017.10.003

[15] Bengtsson AA, Sturfelt G, Truedsson L, Blomberg J, Alm G, Vallin H, et al. Activation of type I interferon system in systemic lupus erythematosus correlates with disease activity but not with antiretroviral antibodies. Lupus. 2000;9(9):664–71. 11199920 10.1191/096120300674499064

[16] Kreis J, Nedic B, Mazur J, Urban M, Schelhorn SE, Grombacher T, et al. RosettaSX: reliable gene expression signature scoring of cancer models and patients. Neoplasia. 2021;23(11):1069–77. 34583245 10.1016/j.neo.2021.08.005 PMC8479477

[17] Grasso CS, Tsoi J, Onyshchenko M, Abril-Rodriguez G, Ross-Macdonald P, Wind-Rotolo M, et al. Conserved Interferon-gamma signaling drives clinical response to immune checkpoint Blockade therapy in melanoma. Cancer Cell. 2020;38(4):500–15. e3. 32916126 10.1016/j.ccell.2020.08.005 PMC7872287

[18] Karachaliou N, Gonzalez-Cao M, Crespo G, Drozdowskyj A, Aldeguer E, Gimenez-Capitan A, et al. Interferon gamma, an important marker of response to immune checkpoint Blockade in non-small cell lung cancer and melanoma patients. Ther Adv Med Oncol. 2018;10:1758834017749748. 29383037 10.1177/1758834017749748 PMC5784541

[19] Mo X, Zhang H, Preston S, Martin K, Zhou B, Vadalia N, et al. Interferon-gamma signaling in melanocytes and melanoma cells regulates expression of CTLA-4. Cancer Res. 2018;78(2):436–50. 29150430 10.1158/0008-5472.CAN-17-1615 PMC5771950

[20] Lee HK, Jung O, Hennighausen L. Activation of Interferon-Stimulated Transcriptomes and ACE2 Isoforms in Human Airway Epithelium Is Curbed by Janus Kinase Inhibitors. Res Sq. 2020:rs.3.rs-119695.

[21] Edgar R, Domrachev M, Lash AE. Gene expression omnibus: NCBI gene expression and hybridization array data repository. Nucleic Acids Res. 2002;30(1):207–10. 11752295 10.1093/nar/30.1.207 PMC99122

[22] Love MI, Huber W, Anders S. Moderated Estimation of fold change and dispersion for RNA-seq data with DESeq2. Genome Biol. 2014;15(12):550. 25516281 10.1186/s13059-014-0550-8 PMC4302049

[23] Ziegler CGK, Allon SJ, Nyquist SK, Mbano IM, Miao VN, Tzouanas CN, et al. SARS-CoV-2 receptor ACE2 is an Interferon-Stimulated gene in human airway epithelial cells and is detected in specific cell subsets across tissues. Cell. 2020;181(5):1016–e3519. 32413319 10.1016/j.cell.2020.04.035 PMC7252096

[24] Jankowski J, Lee HK, Wilflingseder J, Hennighausen L. Interferon-regulated genetic programs and JAK/STAT pathway activate the intronic promoter of the short ACE2 isoform in renal proximal tubules. BioRxiv. 2021:2021.01.15.426908.

[25] Fujiwara Y, Sun Y, Torphy RJ, He J, Yanaga K, Edil BH, et al. Pomalidomide inhibits PD-L1 induction to promote antitumor immunity. Cancer Res. 2018;78(23):6655–65. 30315115 10.1158/0008-5472.CAN-18-1781

[26] Colli ML, Ramos-Rodríguez M, Nakayasu ES, Alvelos MI, Lopes M, Hill JLE, et al. An integrated multi-omics approach identifies the landscape of interferon-α-mediated responses of human pancreatic beta cells. Nat Commun. 2020;11(1):2584. 32444635 10.1038/s41467-020-16327-0 PMC7244579

[27] Rai TS, Glass M, Cole JJ, Rather MI, Marsden M, Neilson M, et al. Histone chaperone HIRA deposits histone H3.3 onto foreign viral DNA and contributes to anti-viral intrinsic immunity. Nucleic Acids Res. 2017;45(20):11673–83. 28981850 10.1093/nar/gkx771 PMC5691367

[28] Devlin JC, Zwack EE, Tang MS, Li Z, Fenyo D, Torres VJ, et al. Distinct features of human myeloid cell cytokine response profiles identify neutrophil activation by cytokines as a prognostic feature during tuberculosis and Cancer. J Immunol. 2020;204(12):3389–99. 32350082 10.4049/jimmunol.1901133 PMC7276940

[29] Goldman MJ, Craft B, Hastie M, Repecka K, McDade F, Kamath A, et al. Visualizing and interpreting cancer genomics data via the Xena platform. Nat Biotechnol. 2020;38(6):675–8. 32444850 10.1038/s41587-020-0546-8 PMC7386072

[30] Kim ST, Cristescu R, Bass AJ, Kim KM, Odegaard JI, Kim K, et al. Comprehensive molecular characterization of clinical responses to PD-1 Inhibition in metastatic gastric cancer. Nat Med. 2018;24(9):1449–58. 30013197 10.1038/s41591-018-0101-z

[31] Van Allen EM, Miao D, Schilling B, Shukla SA, Blank C, Zimmer L, et al. Genomic correlates of response to CTLA-4 Blockade in metastatic melanoma. Science. 2015;350(6257):207–11. 26359337 10.1126/science.aad0095 PMC5054517

[32] Cui C, Xu C, Yang W, Chi Z, Sheng X, Si L, et al. Ratio of the interferon-gamma signature to the immunosuppression signature predicts anti-PD-1 therapy response in melanoma. NPJ Genom Med. 2021;6(1):7. 33542239 10.1038/s41525-021-00169-w PMC7862369

[33] Mariathasan S, Turley SJ, Nickles D, Castiglioni A, Yuen K, Wang Y, et al. TGFbeta attenuates tumour response to PD-L1 Blockade by contributing to exclusion of T cells. Nature. 2018;554(7693):544–8. 29443960 10.1038/nature25501 PMC6028240

[34] Hartoularos GC, Si Y, Zhang F, Kathail P, Lee DS, Ogorodnikov A et al. Reference-free multiplexed single-cell sequencing identifies genetic modifiers of the human immune response. BioRxiv. 2023:2023.05.29.542756.

[35] Stuart T, Butler A, Hoffman P, Hafemeister C, Papalexi E, Mauck WM 3, et al. Comprehensive integration of Single-Cell data. Cell. 2019;177(7):1888–e90221. 31178118 10.1016/j.cell.2019.05.031 PMC6687398

[36] Kartha VK, Duarte FM, Hu Y, Ma S, Chew JG, Lareau CA et al. Functional inference of gene regulation using single-cell multi-omics. Cell Genom. 2022;2(9). 10.1016/j.xgen.2022.100166 PMC9534481 36204155

[37] Aybey B, Zhao S, Brors B, Staub E. Immune cell type signature discovery and random forest classification for analysis of single cell gene expression datasets. Front Immunol. 2023;14:1194745. 37609075 10.3389/fimmu.2023.1194745 PMC10441575

[38] Hao Y, Hao S, Andersen-Nissen E, Mauck WM 3rd, Zheng S, Butler A, et al. Integrated analysis of multimodal single-cell data. Cell. 2021;184(13):3573–e8729. 34062119 10.1016/j.cell.2021.04.048 PMC8238499

[39] Ritchie ME, Phipson B, Wu D, Hu Y, Law CW, Shi W, et al. Limma powers differential expression analyses for RNA-sequencing and microarray studies. Nucleic Acids Res. 2015;43(7):e47. 25605792 10.1093/nar/gkv007 PMC4402510

[40] Rucker G. Network meta-analysis, electrical networks and graph theory. Res Synth Methods. 2012;3(4):312–24. 26053424 10.1002/jrsm.1058

[41] Winter C, Kosch R, Ludlow M, Osterhaus A, Jung K. Network meta-analysis correlates with analysis of merged independent transcriptome expression data. BMC Bioinformatics. 2019;20(1):144. 30876387 10.1186/s12859-019-2705-9 PMC6420731

[42] Balduzzi S, Rücker G, Nikolakopoulou A, Papakonstantinou T, Salanti G, Efthimiou O, et al. Netmeta: an R package for network Meta-Analysis using frequentist methods. J Stat Softw. 2023;106(2):1–40.37138589

[43] Liberzon A, Birger C, Thorvaldsdottir H, Ghandi M, Mesirov JP, Tamayo P. The molecular signatures database (MSigDB) hallmark gene set collection. Cell Syst. 2015;1(6):417–25. 26771021 10.1016/j.cels.2015.12.004 PMC4707969

[44] Bilgic H, Ytterberg SR, Amin S, McNallan KT, Wilson JC, Koeuth T, et al. Interleukin-6 and type I interferon-regulated genes and chemokines mark disease activity in dermatomyositis. Arthritis Rheum. 2009;60(11):3436–46. 19877033 10.1002/art.24936

[45] Feng X, Wu H, Grossman JM, Hanvivadhanakul P, FitzGerald JD, Park GS, et al. Association of increased interferon-inducible gene expression with disease activity and lupus nephritis in patients with systemic lupus erythematosus. Arthritis Rheum. 2006;54(9):2951–62. 16947629 10.1002/art.22044

[46] Rice GI, Forte GM, Szynkiewicz M, Chase DS, Aeby A, Abdel-Hamid MS, et al. Assessment of interferon-related biomarkers in Aicardi-Goutieres syndrome associated with mutations in TREX1, RNASEH2A, RNASEH2B, RNASEH2C, SAMHD1, and ADAR: a case-control study. Lancet Neurol. 2013;12(12):1159–69. 24183309 10.1016/S1474-4422(13)70258-8 PMC4349523

[47] Walsh RJ, Kong SW, Yao Y, Jallal B, Kiener PA, Pinkus JL, et al. Type I interferon-inducible gene expression in blood is present and reflects disease activity in dermatomyositis and polymyositis. Arthritis Rheum. 2007;56(11):3784–92. 17968926 10.1002/art.22928 PMC2443782

[48] Azizi E, Carr AJ, Plitas G, Cornish AE, Konopacki C, Prabhakaran S, et al. Single-Cell map of diverse immune phenotypes in the breast tumor microenvironment. Cell. 2018;174(5):1293–308. e36. 29961579 10.1016/j.cell.2018.05.060 PMC6348010

[49] Platanias LC. Mechanisms of type-I- and type-II-interferon-mediated signalling. Nat Rev Immunol. 2005;5(5):375–86. 15864272 10.1038/nri1604

[50] Sharma P, Retz M, Siefker-Radtke A, Baron A, Necchi A, Bedke J, et al. Nivolumab in metastatic urothelial carcinoma after platinum therapy (CheckMate 275): a multicentre, single-arm, phase 2 trial. Lancet Oncol. 2017;18(3):312–22. 28131785 10.1016/S1470-2045(17)30065-7

[51] Waddell SJ, Popper SJ, Rubins KH, Griffiths MJ, Brown PO, Levin M, et al. Dissecting interferon-induced transcriptional programs in human peripheral blood cells. PLoS ONE. 2010;5(3):e9753. 20339534 10.1371/journal.pone.0009753 PMC2842296

[52] Charoentong P, Finotello F, Angelova M, Mayer C, Efremova M, Rieder D, et al. Pan-cancer Immunogenomic analyses reveal Genotype-Immunophenotype relationships and predictors of response to checkpoint Blockade. Cell Rep. 2017;18(1):248–62. 28052254 10.1016/j.celrep.2016.12.019

[53] Angelova M, Charoentong P, Hackl H, Fischer ML, Snajder R, Krogsdam AM, et al. Characterization of the immunophenotypes and antigenomes of colorectal cancers reveals distinct tumor escape mechanisms and novel targets for immunotherapy. Genome Biol. 2015;16:64. 25853550 10.1186/s13059-015-0620-6 PMC4377852

[54] Becht E, Giraldo NA, Lacroix L, Buttard B, Elarouci N, Petitprez F, et al. Estimating the population abundance of tissue-infiltrating immune and stromal cell populations using gene expression. Genome Biol. 2016;17(1):218. 27765066 10.1186/s13059-016-1070-5 PMC5073889

[55] Bindea G, Mlecnik B, Tosolini M, Kirilovsky A, Waldner M, Obenauf AC, et al. Spatiotemporal dynamics of intratumoral immune cells reveal the immune landscape in human cancer. Immunity. 2013;39(4):782–95. 24138885 10.1016/j.immuni.2013.10.003

[56] Nieto P, Elosua-Bayes M, Trincado JL, Marchese D, Massoni-Badosa R, Salvany M, et al. A single-cell tumor immune atlas for precision oncology. Genome Res. 2021;31(10):1913–26. 34548323 10.1101/gr.273300.120 PMC8494216

[57] Newman AM, Liu CL, Green MR, Gentles AJ, Feng W, Xu Y, et al. Robust enumeration of cell subsets from tissue expression profiles. Nat Methods. 2015;12(5):453–7. 25822800 10.1038/nmeth.3337 PMC4739640

[58] Breheny P, Stromberg A, Lambert J. p-Value histograms: inference and diagnostics. High Throughput. 2018;7(3). 10.3390/ht7030023 PMC6164648 30200313

[59] de Weerd NA, Vivian JP, Nguyen TK, Mangan NE, Gould JA, Braniff SJ, et al. Structural basis of a unique interferon-β signaling axis mediated via the receptor IFNAR1. Nat Immunol. 2013;14(9):901–7. 23872679 10.1038/ni.2667

[60] Thomas C, Moraga I, Levin D, Krutzik PO, Podoplelova Y, Trejo A, et al. Structural linkage between ligand discrimination and receptor activation by type I interferons. Cell. 2011;146(4):621–32. 21854986 10.1016/j.cell.2011.06.048 PMC3166218

[61] Chang YJ, Holtzman MJ, Chen CC. Interferon-gamma-induced epithelial ICAM-1 expression and monocyte adhesion. Involvement of protein kinase C-dependent c-Src tyrosine kinase activation pathway. J Biol Chem. 2002;277(9):7118–26. 11751911 10.1074/jbc.M109924200

[62] Kim BH, Shenoy AR, Kumar P, Bradfield CJ, MacMicking JD. IFN-inducible GTPases in host cell defense. Cell Host Microbe. 2012;12(4):432–44. 23084913 10.1016/j.chom.2012.09.007 PMC3490204

[63] Liu M, Liu J, Hao S, Wu P, Zhang X, Xiao Y, et al. Higher activation of the interferon-gamma signaling pathway in systemic lupus erythematosus patients with a high type I IFN score: relation to disease activity. Clin Rheumatol. 2018;37(10):2675–84. 29774490 10.1007/s10067-018-4138-7

[64] Morrow AN, Schmeisser H, Tsuno T, Zoon KC. A novel role for IFN-stimulated gene factor 3II in IFN-gamma signaling and induction of antiviral activity in human cells. J Immunol. 2011;186(3):1685–93. 21178011 10.4049/jimmunol.1001359 PMC3417313

[65] Pallotta MT, Rossini S, Suvieri C, Coletti A, Orabona C, Macchiarulo A et al. Indoleamine 2,3-dioxygenase 1 (IDO1): an up-to-date overview of an eclectic immunoregulatory enzyme. Febs J. 2021. 10.1111/febs.16086 PMC9786828 34145969

