# Supplementary material for: Expression signatures with specificity for type I and II IFN response and relevance for autoimmune diseases and cancer
Source: J Transl Med. 2025 Jul 3;23:740. doi: 10.1186/s12967-025-06628-7 (PMC12231911; doi:10.1186/s12967-025-06628-7)
Supplement: Supplementary file 1 — Supplementary Material 1 [file 12967_2025_6628_MOESM1_ESM.docx]

# Supplementary Figures

**Supp. Fig. 1. Distribution of SLEDAI scores across three SLE microarray datasets.** Histograms display the range and frequency of SLEDAI values used in downstream correlation analyses.

**Supp. Fig. 2. P-value distributions of differential gene expression tests for each treatment comparison in discovery datasets.** The shape of the distributions with peaks close to zero confirm that there is sufficient signal to identify differentially expressed genes after IFN stimulation in each of the discovery datasets.

**Supp. Fig. 3. FeaturePlots showing mean signature expression scores of IFN Aybey signatures on UMAP plots in IFN-b, IFN-g, and TNF-a stimulation single cell gene expression dataset.** (Upper) UMAP based on our immune cell type specific genes (1) showing different cell types defined by our random forest cell type classification method and different conditions. (Lower) Mean signature score levels are shown for IFN-I and IFN-II Aybey signature from low (gray) to high (red) on UMAP from the upper figure. Each condition is shown in a separate FeaturePlot.

**Supp. Fig. 4. UMAPs based on different IFN gene set collections in IFN-b and IFN-g stimulation single cell gene expression dataset: (A) IFN-I Aybey and IFN-II Aybey genes or (B) published IFN-I and IFN-II genes. Expression data** Expression data of samples from different conditions (control, IFN-b stimulation, and IFN-g stimulation) are used to derive a UMAP based on different IFN gene sets. Each gene set is colored differently, and gene names are shown for our IFN signatures. For published gene sets unique IFN-I or IFN-II genes as well as common genes between those two subtypes are colored. Note that in (A) our IFN-I and IFN-II signature genes are perfectly separated while in (B) for IFN-I/II genes from other published signatures genes do not cluster by annotated IFN type.

**Supp. Fig. 5. IFN-gamma signature activation in PBMCs in Kartha dataset.** Here we show violin plots of IFN-II-Aybey signature score in different immune cell types in PBMCs based on the data of Kartha et al. (2022). Different time points (1h and 6h) are plotted separately. Control and IFNg samples are colored differently. Statistical significance of signature core differences is denoted by asterisks with the following codes: * = p < 0.05, ** = p < 0.01, *** = p < 0.001, **** = p < 0.0001. Note, that the IFN-g signature scores at 1h and 6h after stimulation are significantly higher for each cell type, both in myeloid and non-myeloid cells.

**Supp. Fig. 6. Heatmap for all coherent signatures ranked by covariance to our IFN signatures in TCGA BRCA cohort.** A similar analysis from Kreis et al. is performed using the signatures from Kreis et al. and additional published CD8^+^ T cell and IFN-II signatures (2). Mean signature scores are calculated only for coherent signatures (coherence score > 0.2). Covariance between each of our IFN signatures and coherent signatures are calculated and all coherent signatures are displayed in rows. Mean signature scores are shown from low (blue) to high (red). Covariance values are shown from low (dark blue) to high (orange).

**References:**

1. Aybey B, Zhao S, Brors B, Staub E. Immune cell type signature discovery and random forest classification for analysis of single cell gene expression datasets. Front Immunol. 2023;14:1194745.

2. Kreis J, Nedic B, Mazur J, Urban M, Schelhorn SE, Grombacher T, et al. RosettaSX: Reliable gene expression signature scoring of cancer models and patients. Neoplasia. 2021;23(11):1069-77.
